# A Complex Proteomic Response of the Parasitic Nematode *Anisakis simplex* s.s. to *Escherichia coliLipopolysaccharide*

**DOI:** 10.1016/j.mcpro.2021.100166

**Published:** 2021-10-19

**Authors:** Karol Mierzejewski, Robert Stryiński, Elżbieta Łopieńska-Biernat, Jesús Mateos, Iwona Bogacka, Mónica Carrera

**Affiliations:** 1 Department of Animal Anatomy and Physiology, Faculty of Biology and Biotechnology, University of Warmia and Mazury in Olsztyn, Olsztyn, Poland; 2 Department of Biochemistry, Faculty of Biology and Biotechnology, University of Warmia and Mazury in Olsztyn, Olsztyn, Poland; 3 Galapagos NV, Mechelen, Belgium; 4 Department of Food Technology, Marine Research Institute (IIM), Spanish National Research Council (CSIC), Vigo, Spain

**Keywords:** *Anisakis simplex*, lipopolysaccharide, parasite–bacteria interrelationship, oxidative stress, LC–MS/MS, ABTS, 2,2'-azinobis-(3-ethylbenzothiazoline-6-sulfonic acid), BP, biological process, cDNA, complementary DNA, DRP, differentially regulated protein, ERAP1, endoplasmic reticulum aminopeptidase 1, FC, fold change, *gdf 11*, growth/differentiation factor 11, GO, Gene Ontology, GST, glutathione-*S*-transferase, HSD, honest significant difference, IL, interleukin, LPS, lipopolysaccharide, MF, molecular function, MHC, major histocompatibility complex, MIF, macrophage migration inhibitory factor, PRDX, peroxiredoxin, ROS, reactive oxygen species, RQ, relative quantification, TGF-β, transforming growth factor β, TMT, tandem mass tag

## Abstract

Helminths are masters at manipulating host's immune response. Especially, parasitic nematodes have evolved strategies that allow them to evade, suppress, or modulate host's immune response to persist and spread in the host's organism. While the immunomodulatory effects of nematodes on their hosts are studied with a great commitment, very little is known about nematodes' own immune system, immune response to their pathogens, and interactions between parasites and bacteria in the host's organism. To illustrate the response of the parasitic nematode *Anisakis simplex* s.s. during simulated interaction with *Escherichia coli*, different concentrations of lipopolysaccharide (LPS) were used, and the proteomic analysis with isobaric mass tags for relative and absolute quantification (tandem mass tag–based LC–MS/MS) was performed. In addition, gene expression and biochemical analyses of selected markers of oxidative stress were determined. The results revealed 1148 proteins in a group of which 115 were identified as differentially regulated proteins, for example, peroxiredoxin, thioredoxin, and macrophage migration inhibitory factor. Gene Ontology annotation and Reactome pathway analysis indicated that metabolic pathways related to catalytic activity, oxidation–reduction processes, antioxidant activity, response to stress, and innate immune system were the most common, in which differentially regulated proteins were involved. Further biochemical analyses let us confirm that the LPS induced the oxidative stress response, which plays a key role in the innate immunity of parasitic nematodes. Our findings, to our knowledge, indicate for the first time, the complexity of the interaction of parasitic nematode, *A. simplex* s.s. with bacterial LPS, which mimics the coexistence of helminth and gut bacteria in the host. The simulation of this crosstalk led us to conclude that the obtained results could be hugely valuable in the integrated systems biology approach to describe a relationship between parasite, host, and its commensal bacteria.

## Highlights

• LPS induces a complex proteomic response in a parasitic nematode *Anisakis simplex* s.s.
• LPS induces oxidative stress in *A. simplex* s.s.
• Peroxiredoxins are strongly involved in the response to LPS in *A. simplex* s.s.
• This experimental setup mimics the coexistence of parasite and bacteria in the host.

## Graphical Abstract

**Figure:**
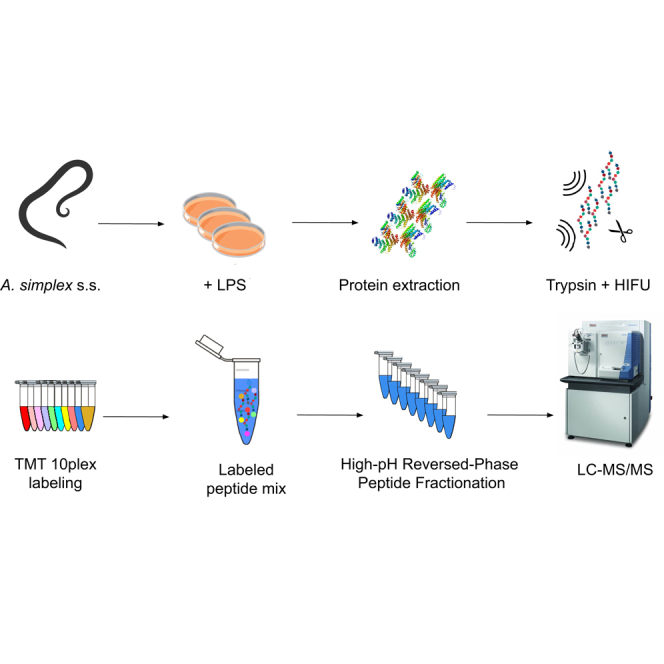

## In Brief

*Anisakis simplex* s.s. is a seafood-borne parasite that is involved in human infections. This experimental setup mimics the coexistence of helminth and gut bacteria in the host. Describing the mechanisms of the proteomic response of *A. simplex* to LPS can contribute to better understanding the parasite biology and more effective treatment of anisakiasis. Our findings indicate the complexity of the proteomic response of this parasite to LPS. Obtained results are valuable in planning future strategies for studying helminths.

*Anisakis simplex* s.s. is one of the seafood-borne parasitic nematodes, which is commonly involved in human infections (1). The disease caused by the Anisakis genus is called anisakiasis (2, 3). Humans, as accidental hosts, can be infected by third-stage larvae (L3) present in raw or undercooked fish or cephalopods (2). The infection with *A. simplex* s.s. can cause gastrointestinal symptoms or mild to severe allergic reactions (4, 5). During recent years, progress in the food industry and its globalization have changed the eating habits of people all over the world (1). According to the European Food Safety Authority, nematode infections from food are ubiquitous in Europe. In addition, an expert panel has classified *A. simplex* as a biohazardous organism (6, 7). Moreover, climate change and increasing water temperatures have caused *A. simplex* s.s. to expand its range, allowing this species to occur in seas and oceans, where it was not previously found (8). Anisakiasis is a relatively new and rapidly spreading zoonosis that poses a significant threat to humans and animals. The disease causes economic losses in the fisheries sector by undermining consumer confidence and reducing demand for potentially infected fish. At a time when travel is widespread and international trade is rapidly increasing, anisakiasis is becoming a global problem. The growing popularity of exotic dishes prepared from raw fish and cephalopods and the widespread practice of undercooking seafood are also contributing to the spread of the disease (9, 10, 11, 12). All these could be a reason for the increasing number of anisakiasis cases. The incidence of anisakiasis continues to increase, and 20,000 new cases were reported in 2010. According to the quantitative risk assessment model, the prevalence of anisakiasis in Europe will increase to 7500 to 8500 cases per year (13).

Recent studies reported that the presence of helminth infection alters the composition of the bacterial intestinal microbiota and, conversely, that the presence and composition of the bacterial microbiota affect helminth colonization and persistence within mammalian hosts (14). The crosstalk between helminths and the bacteria of the host and their interactions are largely unknown. Currently, the concept of parasite–bacteria interactions in vertebrate bodies is of great interest. These interactions can be neutral, harmful, or have beneficial effects (15, 16). In general, intestinal helminths increase the expression of antimicrobial peptides in the digestive tract, such as angiogenin 4 after infection with *Trichuris muris* and C-type lectin RegIIIγ following *Heligmosomoides polygyrus* infection in mice (17, 18). This kind of interaction can lead to changes in the composition of the microbiota after helminth infetion. On the other hand, the bacterial microbiota-specific immune response during parasite infection may reduce the immune response to helminth antigens. Alternatively, the microbiota can increase mucosal or systemic immunity to parasitic infections by changing innate or adaptive immunity (19).

Compared with another species, relatively little is known about the immune responses in the *A. simplex* nematode. In common with other invertebrates, nematodes defense against pathogens that rely on innate immune response (20). During defense processes, the immune system triggers the formation of large amounts of reactive oxygen species (ROS) like superoxide anion (O_2_), hydroxyl radical (OH), and hydrogen peroxide (H_2_O_2_), which are accumulated in the cells (21). ROS act as signaling molecules and represent an efficient and highly conserved effector mechanism to eliminate pathogens in animals and plants (22). In a free-living nematode, *Caenorhabditis elegans*, ROS can activate protective cellular mechanisms to promote longevity, pathogen defense responses, and wound healing (23). Moreover, *C. elegans* infected with *Enterococcus faecalis* produce ROS *via* the dual oxidase Duox1/BLI-3 (present in the intestine), which represents a protective antimicrobial response (24, 25). Nematodes can be also exposed to ROS released from immune effector cells like macrophages, neutrophils, and eosinophils produced within their vertebrate hosts (26). Despite the fact that ROS plays a crucial role in defense against bacterial infection, their overexpression or production by the parasite hosts' require mechanisms by which nematodes can protect themselves (26). Therefore, parasitic nematodes have developed effective antioxidant defense systems, including enzymes that deal with the ROS, like superoxide dismutase, catalase, and peroxiredoxins (PRDXs), to detoxify and regulate intracellular homeostasis (26).

Although many of those mechanisms have been described on intestinal nematodes when considering them as pathogenic to their hosts (mammalian host–parasite relationship), the global overview on the molecular processes occurring in the helminths during the coexistence with bacteria and their influence on parasites have not been fully characterized (parasite–bacteria interrelationship). The shared effects of bacteria and helminths include suppression of the host immunity to permit their survival. Therefore, they have common strategies that include the activation of regulatory T cells by some bacteria such as *Bacteroides fragilis* and *Lactobacillus* spp. and by some parasites including *H. polygyrus* and *Strongyloides ratti* (14). The mammalian immune system has learned to distinguish which microorganisms reject or accept. Moreover, both commensal bacteria and intestinal helminths have developed similar strategies of modulating host immunity. Interestingly, they have developed a surprising degree of dialog with a common goal of establishing new homeostasis in the host intestinal tract to survive (14, 15, 27). Therefore, it is not known whether the digestive tract bacteria should be treated as pathogenic to intestinal parasites or as coexisting organisms whose metabolic pathways are to some extent specifically integrated (28).

To study the interaction of *A. simplex* s.s. with the human microbiome, we decided to treat the larvae with the lipopolysaccharide (LPS) of *Escherichia coli*, a bacterium found in the human gut. The results by Guardone *et al.* (29) showed that gastric and intestinal localizations of Anisakis larvae during infection had very similar frequencies, with only a slightly higher frequency of gastric lesions. Also, in a retrospective case series study conducted in Tokyo, Japan, 47% of patients had gastric anisakiasis and 53% had small intestinal anisakiasis (30). However, other authors note that in Japan the acute gastric form predominates (95%), whereas in Europe, the chronic intestinal form seems to be more common (31, 32). For all these reasons, we decided to try to explain possible interactions of *A. simplex* s.s. with a representative of the human gut microbiome, namely *E. coli*, using a rapidly developing branch of biology, proteomics.

In the last 2 decades, proteomics has become a powerful tool for describing dynamically changing biological systems (33). Proteomics methods were not only used for the identification and quantification of the protein composition of cells, tissue sections, and whole organisms at a certain time, collectively termed “the proteome” but also to describe protein modifications and interactions (34). Proteomics in the studies on parasitic species has been widely used (1, 35). Moreover, proteomics on *A. simplex* s.s. was previously performed (36, 37) but never in the context of parasite–bacteria interactions.

Accordingly, in this work, using the tandem mass tag (TMT)-based quantitative proteomics method based on TMT-isobaric mass tag labeling and LC–MS/MS analysis in an LTQ-Orbitrap Elite mass spectrometer, we present for the first time the proteome profiling of *A. simplex* s.s. L3 larvae stimulated by bacterial LPS from *E. coli*, an *in vitro* model of the interplay between parasites and bacteria.

## Experimental Procedures

### Anisakis simplex

All experiments were performed on the alive L3 larvae of *A. simplex* s.s. from Baltic herring (*Clupea harengus membras*) caught in the coastal waters of the southern Baltic Sea. All impurities were removed from the harvested larvae. Then, the larvae were washed several times in a sterile 0.9% NaCl and stored at 4 °C until further analyses. At the beginning of the study, five of the larvae were subjected to the taxonomic identification by Anis Sensitive Sniper Real-Time PCR kit (A & A Biotechnology, Gdynia, Poland) as described before (36).

### Preliminary Study

#### *In Vitro* Culture With LPS

To choose the optimal LPS concentrations for an *in vitro* model of parasite–bacteria interaction, the preliminary culture of *A. simplex* s.s. L3 larvae was conducted under anerobic conditions (37 °C, 5% CO_2_) in the presence of LPS isolated from *E. coli* (Sigma–Aldrich; L8274-10MG) at different concentrations: 0.05, 0.1, 0.15, 0.2, 0.4, 0.6, 0.8, 1, and 2 μg/ml for 12 h. The procedure of *in vitro* culture was carried out as described previously by Iglesias *et al.* (38) using the reagents as described before (36, 39). Three *A. simplex* s.s. L3 larvae were placed in each well of the 6-well culture plates (BD Biosciences) (27 in total). The parasites without LPS were cultured as a control (three larvae × three wells; nine in total).

#### Real-Time PCR Analysis of Growth/Differentiation Factor 11

It was reported before that homologs of human transforming growth factor β (TGF-β) from an intestinal helminths mimics host's TGF-β biological and functional properties and induces potently suppressive regulatory T cells engaged in inflammatory process (40). In *A. simplex* s.s., such homolog of human TGF-β, that is, growth/differentiation factor 11 (*gdf 11*), was previously identified by Łopieńska-Biernat *et al.* (41) during genome-wide analysis of this species. To determine the *gdf 11* mRNA level in *A. simplex* s.s., the real-time PCR was performed. The primers for the *gdf 11* were designed using the Primer3Plus software (42) (ELIXIR) based on the sequence deposited in GenBank (MF069104.1) and listed in supplemental Table S1. The actin was used as the endogenous reference gene (43). The reaction was performed as described before (36). In brief, the total RNA of the larvae was isolated using a Total RNA Mini Kit (A & A Biotechnology), and complementary DNA (cDNA) was obtained using TranScriba Kit (A & A Biotechnology) according to the manufacturer's protocols. The real-time PCR mixture contained 1 μl of cDNA, 5 μl of 2× SYBR RT PCR MIX SYBR B (A & A Biotechnology), 0.25 μl of each primer, 0.25 μl of Rox Reference Dye II (A & A Biotechnology), and 3.25 μl of nuclease-free water to a final volume of 10 μl. The reactions were performed in six replicates on a 7500 Fast Real-Time PCR thermocycler (Applied Biosystems). The relative expression, presented as the fold change (FC) relative to the untreated control, as well as normalized to an endogenous reference gene (actin) (relative quantification [RQ] = 1), was calculated using the comparative Pfaffl method (44). The data were expressed as means ± SDs. Statistical analysis was performed using one-way ANOVA in Prism 8 software (GraphPad Software, Inc). Differences between means were assessed by Tukey's multiple comparison test. *p* Values were considered statistically significant: 0.0332 (∗), 0.0021 (∗∗), 0.0002 (∗∗∗), and <0.0001 (∗∗∗∗).

### *In Vitro* Culture of *A. simplex* With LPS

**Fig. 1:**
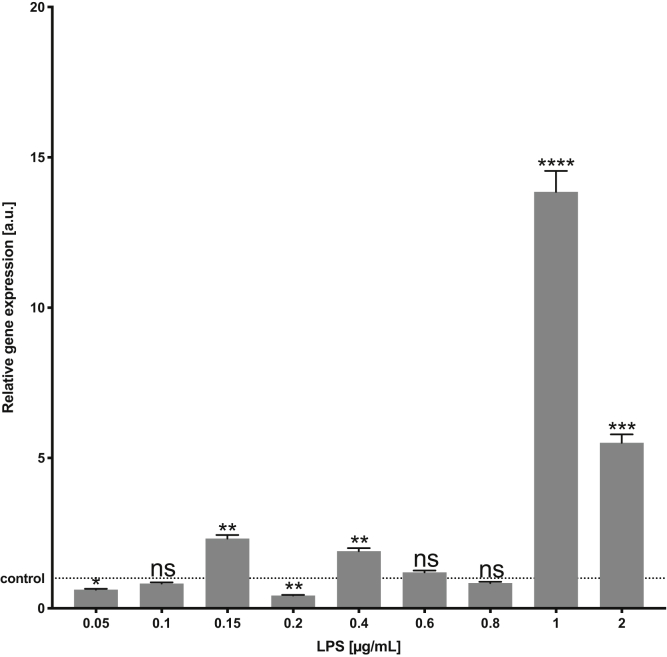
**The expression of *gdf 11* in *Anisakis simplex* s.s. L3 larvae exposed *in vitro* (12 h) to different LPS concentrations (0.05, 0.1, 0.15, 0.2, 0.4, 0.6, 0.8, 1, and 2 μg/ml).** Depicted values indicate means of six replicates ± SD. The data were presented as the fold change in gene expression normalized to an endogenous reference gene and relative to the untreated control (relative quantification [RQ] = 1). *p* Values were considered statistically significant, where 0.0332 (∗), 0.0021 (∗∗), 0.0002 (∗∗∗), and <0.0001 (∗∗∗∗). LPS, lipopolysaccharide.

Based on the results of the preliminary study (Fig. 1), for further experiments, two concentrations of LPS (0.2 μg/ml—the lowest expression of *gdf 11* and 1 μg/ml—the highest expression of *gdf 11*) were selected to induce the proteomic response in *A. simplex* s.s. The *in vitro* culture of the *A. simplex* s.s. L3 larvae with LPS was performed as described in “In Vitro Culture With LPS” section. About 20 larvae were placed in each well of the culture plate (BD Biosciences) and incubated for 12 h with LPS (two concentrations × 20 larvae × three replicates; 120 larvae in total). The parasites without LPS were cultured as a control (20 larvae × three replicates).

### Protein Extraction, Preparation, and Analyses

#### Protein Extraction

Proteins were extracted as described before by Stryiński *et al.* (39). After *in vitro* culture, the parasites (ten larvae × three replicates of each sample: 0.2 μg/ml abbreviated further as “LPS 0.2” and 1 μg/ml abbreviated further as “LPS 1” and control) were crushed manually with a sterile plastic pestle in 2 ml centrifuge tubes. Then, protein extraction was performed in 1.5 ml of lysis buffer (60 mM Tris–HCl, pH 7.5, 1% lauryl maltoside, 5 mM PMSF, and 1% DTT). The protein concentration was quantified using the bicinchoninic acid method (Pierce BCA Protein Assay Kit; Thermo Fisher Scientific) according to the manufacturer's protocol. Then, the SDS-PAGE electrophoresis was performed as a control step to determine whether the protein extraction was done correctly (39). Running conditions were 80 V for the first 20 min and then 120 V until the end of the electrophoresis. Gels were silver stained using the Pierce Silver Stain for Mass Spectrometry kit (Thermo Fisher Scientific) according to the manufacturer's protocol (supplemental Fig. S1*A*). A total of 100 μg of the protein from each sample (nine samples in total) was transferred into new tubes, and methanol/chloroform precipitation was performed as described by Carrera *et al.* (45). Then, ultrafast tryptic digestion with the simultaneous application of high-intensity focused ultrasound was carried out, as described previously by Stryiński *et al.* (37, 39).

#### TMT Labeling and Reversed-Phase Fractionation

The TMT 10-plex isobaric label reagents (0.8 mg; Thermo Fisher Scientific) were resuspended in 41 μl of anhydrous acetonitrile and added to 100 μg of protein digest, as described by Stryiński *et al.* (39). Within the experiment, samples were labeled with TMT10-plex in triplicate (LPS 0.2: 128N, 128C, and 129N; LPS 1: 129C, 130N, and 130C; without LPS as a control: 126, 127N, and 127C). Samples were combined in a new tube at equal amounts according to the manufacturer's instructions. The TMT-labeled peptide concentration was measured using a Pierce Quantitative Colorimetric Peptide Assay (Thermo Fisher Scientific) according to the manufacturer's instructions. To increase the number of peptide identifications, eliminate the interference from coisolated ions and achieve results comparable to the MS3-based methods (46), the combined sample was fractionated using a Pierce High-pH Reversed-Phase Peptide Fractionation Kit (Thermo Fisher Scientific) following the manufacturer's instructions (supplemental Figs. S1*C* and S2–S4). The peptide concentration in each fraction was determined by colorimetric analysis using the Quantitative Colorimetric Peptide Assay (Thermo Fisher Scientific) following the manufacturer's instructions. Then, fractions were evaporated to dryness using vacuum centrifugation (SpeedVac concentrator; Thermo Fisher Scientific). The samples (eight fractions plus the wash and flow throughput) were stored at −80 °C until further analysis.

#### LC–MS/MS Analysis and Data Processing

Peptide fractions were acidified with 0.1% formic acid, cleaned on a C_18_ MicroSpin column (The Nest Group), and analyzed by LC–MS/MS using a Proxeon EASY-nLC II liquid chromatography system (Thermo Fisher Scientific) coupled to an LTQ-Orbitrap Elite mass spectrometer (Thermo Fisher Scientific). Peptide separation (1 μg) was done as described by Stryiński *et al.* (37, 39).

All acquired MS/MS spectra were analyzed using SEQUEST-HT (Proteome Discoverer 2.4 package; Thermo Fisher Scientific) against a custom-made database containing protein entries for *A. simplex* plus “Nematoda,” available in the UniProt/TrEMBL database (downloaded November 2019; 1,847,926 entries). The following restrictions were used: full tryptic cleavage with up to two missed cleavage sites and tolerances of 10 ppm for parent ions and 0.06 Da for MS/MS fragment ions. TMT labeling (+229.163 Da on N termini and lysine residues) and carbamidomethylation of cysteine (+57.021 Da) were set as fixed modifications. The permissible variable modifications were methionine oxidation (+15.994 Da), acetylation (+42.011 Da) of the N terminus of the protein, and deamidation (+0.984 Da) of asparagine and glutamine. Moreover, searching parameters included four maximal dynamic modification sites (37).

#### Statistical Analysis

The results were subjected to statistical analysis to determine the peptide false discovery rate using a decoy database and the Target Decoy PSM Validator algorithm (47). The false discovery rate was kept below 1%, and for further analysis, only proteins meeting selected criteria were submitted: (a) proteins quantified with at least two unique peptides (supplemental Fig. S1*B*), (b) proteins with different protein IDs, (c) proteins matched for organisms from Rhabditida order, and (d) proteins classified as characterized. RQ was performed using the Quantification Mode and normalization against total peptide amount (Proteome Discoverer 2.4 package).

After RQ, several filters were applied to obtain the final list of differentially regulated proteins (DRPs): (a) at least a one FC in normalized ratios of control *versus* LPS 0.2, control *versus* LPS 1, and LPS 1 *versus* LPS 0.2 and (b) ANOVA on ranks and Tukey honest significant difference (HSD) post hoc test (*p* ≤ 0.05).

#### Functional Categories of Identified Proteins

The final list of proteins obtained after RQ (1148) was classified into three different categories of Gene Ontology (GO): biological processes (BPs), cell components, and molecular functions (MFs). GO analysis was performed using g:GOSt, the core of the g:Profiler (ELIXIR) that performs statistical enrichment analysis (48) (https://biit.cs.ut.ee/gprofiler/gost). The g:GOSt web-based tool applied an overrepresentation test controlled with the g:SCS algorithm. The significantly enriched functional GO categories were reported by comparing the input data with the background of GO annotations for parasite-specific data from WormBase ParaSite (*A. simplex* PRJEB496).

#### Network Analysis

Network analysis was performed by submitting the DRP dataset to Cytoscape (version 3.8.0.; NIGMS), a software platform for visualizing complex networks, and analyzed by stringApp (version 1.5.1.) (49). Interactions have been identified by comparing the input data with the background of the *C. elegans*, the phylogenetically closest nematode available in the stringApp database. The network was limited only to the proteins that have at least one interaction with other proteins submitted to the analysis. The analyzed pathways were classified with the use of Reactome biological pathways database (50) based on *C. elegans* classification.

### Biochemical Analyses

#### Extract Preparation for Biochemical Analyzes

The extract of the larvae after *in vitro* culture (ten larvae × three replicates of each sample: LPS 0.2, LPS 1, and control) for biochemical analyses was prepared by mechanical homogenization (Omni tissue Homogenizer, Omni, Inc) in sterile PBS (pH = 7.4). The extracts were centrifugated in 4 °C by 15 min (5000*g*), and supernatants were transferred into new tubes in amount of 300 μl. The protein concentration was determined using the bicinchoninic acid method (Pierce BCA Protein Assay Kit; Thermo Fisher Scientific) according to the manufacturer's protocol.

#### PRDX Activity Assay

Reduction of peroxide (H_2_O_2_) was measured using the ferrous oxidation–xylenol orange assay (Pierce quantitative peroxide assay kit; Thermo Fisher Scientific). Reactions were performed at 22 °C and initiated by mixing 20 μl of previously prepared supernatants with 200 μl of working reagent. The control reactions were run in parallel using supernatants from control samples. After incubation for 30 min, absorbance was measured at 562 nm on a microplate reader (Asys UVM340; Biochrom). Peroxide standards (4–1000 μM) were included in each assay and used to calculate the quantities of H_2_O_2_. Three technical replicates out of each biological replicate (3 × LPS 0.2, 3 × LPS 1, and 3 × control) were performed. PRDX activity was described based on quantities of H_2_O_2_, where the lower quantity of remaining H_2_O_2_ indicates the higher activity of PRDXs compared with the control.

#### Antioxidant Capacity

Total antioxidant capacity was analyzed by the improved 2,2'-azinobis-(3-ethylbenzothiazoline-6-sulfonic acid) (ABTS) radical cation decolorization assay according to Re *et al.* (51). The preformed radical monocation of ABTS∗+ is generated by oxidation of ABTS with potassium persulfate and is reduced in the presence of such hydrogen-donating antioxidants. Three technical replicates out of each biological replicate (3 × LPS 0.2, 3 × LPS 1, and 3 × control) were performed. The results were calculated as Trolox (a water-soluble analog of vitamin E) equivalents per liter.

#### GSH Content

GSH content was measured according to the Ellman method (52), which was involved in nonenzymatic reduction of 5,5′-dithiobis (2-nitrobenzoic acid), by GSH. The 50 μl of previously prepared larvae supernatants were deproteinized with 10% trichloroacetic acid at the 1:12 ratio before analysis. Three technical replicates out of each biological replicate (3 × LPS 0.2, 3 × LPS 1, and 3 × control) were performed. GSH content was calculated based on the standard curve plotted for serial dilution of 10 mM GSH solution.

#### Glutathione *S*-Transferase Activity

The glutathione-*S*-transferase (GST) activity was determined using the Rice-Evans method (53). Enzyme activity was calculated based on the millimolar absorption coefficient (9.6 mmol^−1^/cm^−1^) for GSH conjugate formed from 1-chloro-2,4-dinitrobenzene. Three technical replicates out of each biological replicate (3 × LPS 0.2, 3 × LPS 1, and 3 × control) were performed. The GST activity was converted into the arbitrary units per 1 mg of protein.

#### Statistical Analysis for Biochemical Analyses

Statistical analyses for the obtained results were performed using ordinary one-way ANOVA (with Tukey post hoc test) in Prism 8 software (GraphPad Software, Inc). *p* Values were considered statistically significant, where 0.0332 (∗), 0.0021 (∗∗), 0.0002 (∗∗∗), and <0.0001 (∗∗∗∗).

### Real-Time PCR Analysis

The proteins involved in the oxidative stress, classified after LC–MS/MS analysis, were chosen to determine the mRNA expression in the L3 larvae of *A. simplex* s.s. by real-time PCR. The *prdx-1* gene was selected, based on its stable protein abundance (not modulated). The *thioredoxin domain–containing protein* and *thioredoxin domain–containing protein 12* as well as *prdx-3* genes were selected based on the downregulation of its proteins caused by LPS, whereas *prdx-2* was selected because of the upregulation of PRDX-2 protein in one of LPS-treated *versus* untreated control. The primers for the selected five genes were designed using the Primer3Plus software (42) (ELIXIR) and listed in supplemental Table S1. The protocol to perform real-time PCR and statistical analysis was described in “Real-Time PCR Analysis of Growth/Differentiation Factor 11” section. The cDNA to perform the reaction was obtained during the preliminary study.

### Experimental Design and Statistical Rationale

To test the three different conditions (control, and two LPS concentrations: 0.2 μg/ml abbreviated as “LPS 0.2” and 1 μg/ml abbreviated as “LPS 1”), three biological replicates of each *in vitro* culture (condition) of *A. simplex* s.s. were performed (nine in total). About 20 larvae were placed in each well of the culture plate (three conditions × 20 larvae × three biological replicates; 180 larvae in total).

After *in vitro* incubation, each sample for LC–MS/MS analysis was prepared out of ten larvae (× three conditions × three biological replicates). Within the experiment, samples were labeled with TMT10-plex in triplicate (LPS 0.2: 128N, 128C, 129N; LPS 1: 129C, 130N, and 130C; and without LPS as a control: 126, 127N, and 127C). The Kruskal–Wallis one-way ANOVA on ranks and Tukey HSD post hoc test (*p* ≤ 0.05) was performed to identify proteins with significant higher or lower abundance.

The second part of the larvae from each *in vitro* culture (three conditions × ten larvae × three biological replicates) was used to prepare extract of the larvae for the biochemical analyses. For each biochemical analysis, three technical replicates out of each biological replicate (3 × LPS 0.2, 3 × LPS 1, and 3 × control) were performed. The data were expressed as means ± SDs. Statistical analysis was performed using one-way ANOVA. Differences between means were assessed by Tukey's multiple comparison test. *p* Values were considered statistically significant, where 0.0332 (∗), 0.0021 (∗∗), 0.0002 (∗∗∗), and <0.0001 (∗∗∗∗).

## Results

### Preliminary Study to Determine the Doses of LPS

The expression of the *gdf 11* in L3 larvae of *A. simplex* s.s. varied in the presence of different doses of LPS (Fig. 1). The LPS doses—0.2 and 1 μg/ml—were selected, based on their significant impact on the lowest and highest expression of *gdf 11*, respectively, when compared with the control.

### Specific Proteome Changes in *Anisakis simples* s.s. After LPS Treatment

As a result of the LC–MS/MS analysis, we identified in a total 4222 master proteins (supplemental File S1). These data were further processed according to the selected criteria: (a) proteins quantified with at least two unique peptides (2157 proteins left), (b) proteins with different protein IDs (1744 proteins left), (c) proteins matched only for organisms from Rhabditida order (1728 proteins left), and (d) characterized proteins (1148 proteins left). For further analysis, we used in total 1148 proteins (supplemental File S2).

**Fig. 2:**
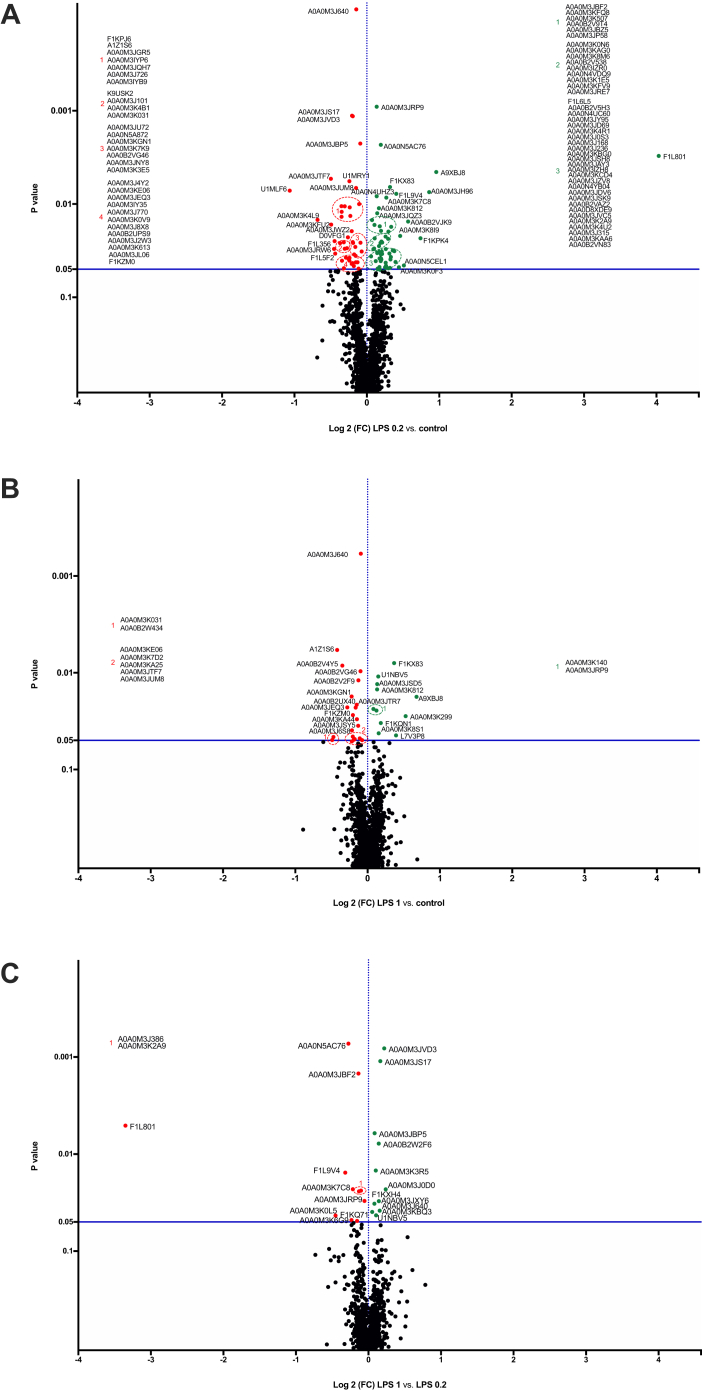
**Detailed visualization of differentially regulated proteins of L3 larvae of *Anisakis simplex* s.s. proteome during LPS-induced inflammation.** Volcano-plot representations of statistical analysis of performed comparisons: (*A*) 0.2 μg/ml of LPS *versus* control, (*B*) 1 μg/ml of LPS *versus* control, and (*C*) 1 μg/ml of LPS *versus* 0.2 μg/ml of LPS. The most upregulated proteins were toward the right (*green*), the most downregulated proteins were toward the left (*red*), and out of them, the most statistically significant proteins were toward the top. The legend for protein IDs is presented in supplemental File S2. LPS, lipopolysaccharide.

After RQ, next several filters were applied to obtain the final list of DRPs: (a) at least a one FC in normalized ratios of control *versus* LPS 0.2, control *versus* LPS 1, and LPS 1 *versus* LPS 0.2; (b) ANOVA and Tukey HSD post hoc test (*p* ≤ 0.05) (supplemental File S2). Volcano plot representations of DRPs are shown in Figure 2, *A*–*C*. In all presented volcano plots, the most upregulated proteins were toward the right (*green*), the most downregulated proteins were toward the left (*red*), and out of them, the most statistically significant proteins were toward the top.

The response of *A. simplex* s.s. to the LPS-induced inflammation was different, depending on the dose of LPS. Proteomic analysis indicated that 115 proteins were differentially regulated in the LPS-treated larvae compared with the control, of which 54 were upregulated and 45 were downregulated in the larvae cultured with 0.2 μg/ml of LPS, whereas 11 proteins were upregulated and 19 were downregulated in the larvae cultured with 1 μg/ml of LPS (FC = 1.0; *p* ≤ 0.05) (Fig. 2, *A*–*C* and supplemental Tables S2 and S3).

**Fig. 3:**
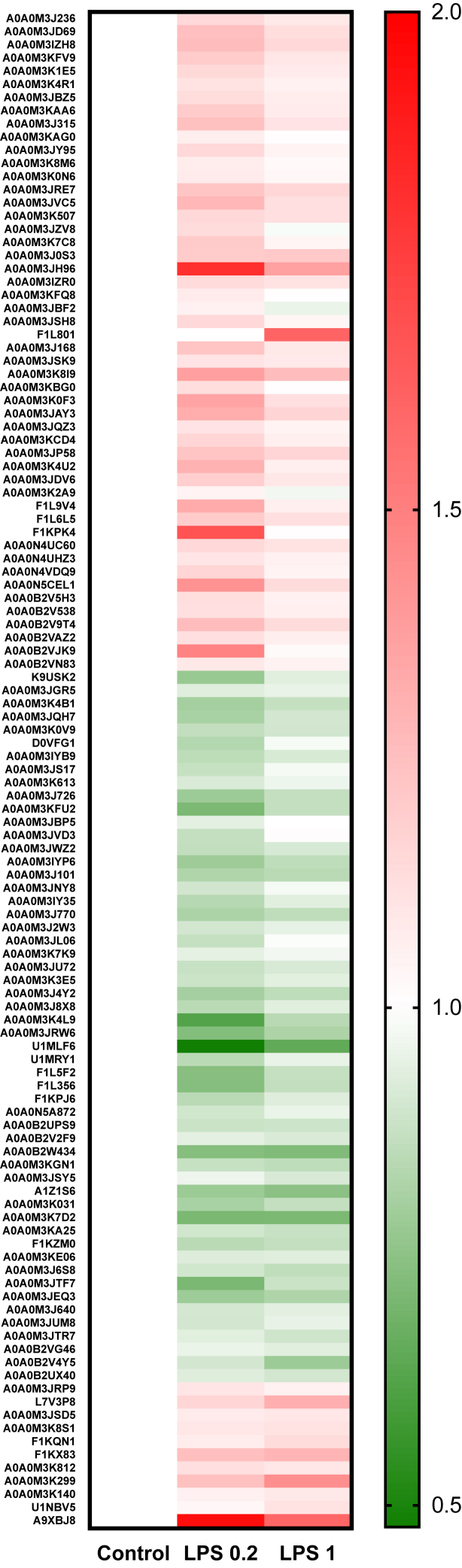
**Visualization of the DRPs statistically significant (adjusted *p* < 0.05) in two comparisons (control *versus* LPS 0.2, control *versus* LPS 1).***Red* (upregulated) and *green* (downregulated) *squares* describe increased and decreased expression in the compared groups, respectively. DRP, differentially regulated protein; LPS, lipopolysaccharide.

In the group of upregulated proteins (supplemental Tables S2 and S3), we identified those with catalytic activity including oxidoreductases (*i.e.*, dihydroorotate dehydrogenase, inosine-5′-monophosphate dehydrogenase, pyruvate dehydrogenase E1 component subunit alpha, or glutamate dehydrogenase) and hydrolases (*i.e.*, Tr-type G domain–containing protein, 1,4-alpha-glucan branching enzyme, or MPN domain–containing protein). Among the proteins regulating antioxidant response, we found PRDX 2, thioredoxin GSH reductase, and 60S ribosomal protein L31. In the group of downregulated proteins (supplemental Tables S2 and S3), we identified other antioxidant proteins such as PRDX 3 and two thioredoxin domain–containing proteins. In the cohort of DRPs, we also noticed proteins that take part in immune response, for example, cathepsin D, galectin, macrophage migration inhibitor factor, twitchin, or tetraspanin. Additional graphical representation of differences in the abundance of DRPs between the control and LPS-treated larvae were shown in Figure 3.

### DRPs Are Associated With Diverse Biological Pathways

**Fig. 4:**
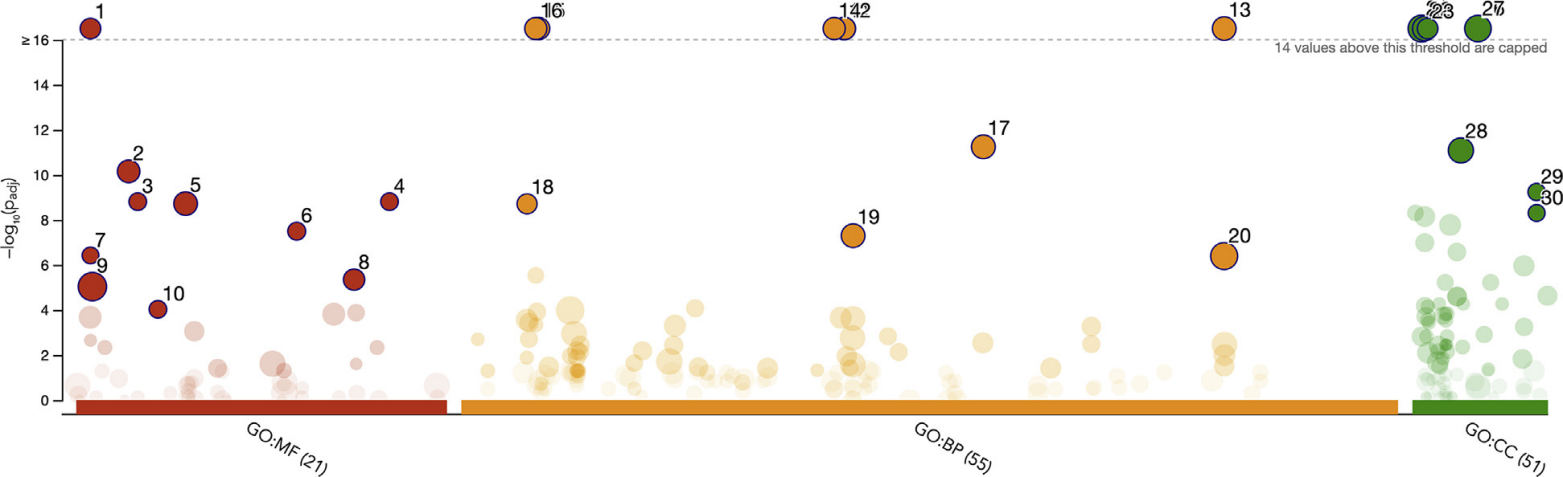
**Manhattan plot that illustrates the results of GO analysis.** The functional terms are grouped and color coded by data sources, that is, molecular function (MF; in *red*), biological processes (BPs; in *orange*), and cellular components (CCs; in *green*). About ten top subcategories from each category are marked by a number and described in Table 1. Detailed representation and annotation of all proteins submitted to the analysis can be found in supplemental File S3. GO, Gene Ontology.

The 1148 proteins were assigned to functional ontology annotations. GO analysis divided the input proteins into three different categories: MF (21 different functions), BPs (55 different processes), and cellular components (51 different components) (Fig. 4; supplemental File S3). The top ten subcategories (a = 0.05) assigned for each of three main GO annotations are presented in Table 1. The functions assigned to the MF category, with predominant activity, were *structural constituent of ribosome* and *structural molecule activity* (GO: 000373548 proteins and GO: 000519862 proteins), *translation factor activity and regulator activity* (GO: 000813519 proteins and GO: 009007919 proteins), and *oxidoreductase activity* (GO: 0016491; 79 proteins) (Fig. 4 and Table 1). In the BP category, most of the proteins were involved in the *cellular amide biosynthetic process* (GO: 0043603, 90 proteins), as well as in the *organonitrogen compound biosynthetic process* (GO: 1901566; 114 proteins), and *oxidation–reduction process* (GO: 0055114, 94 proteins) (Fig. 4 and Table 1). The distribution of the identified proteins according to their abundance in the cellular components was associated with *intracellular structures* (GO: 0005622, 241 proteins), most of them were predicted to be localized in the *cytoplasm* (GO: 0005737, 133 proteins) and *ribosome* (GO: 0005840, 49 proteins) (Fig. 4 and Table 1) (detailed description in supplemental File S3).

**Table 1:** Ten top subcategories from each GO category obtained in g:Profiler

Source	Term name	Term ID	Adjusted *p*	Term size	Input data size
GO:MF	Structural constituent of ribosome	GO: 0003735	1.6704069127181226e-18	138	48
GO:MF	Structural molecule activity	GO: 0005198	6.99280176265433e-11	312	62
GO:MF	Translation factor activity, RNA binding	GO: 0008135	1.54007297998749e-9	40	19
GO:MF	Translation regulator activity, nucleic acid binding	GO: 0090079	1.54007297998749e-9	40	19
GO:MF	Oxidoreductase activity	GO: 0016491	1.879772571633762e-9	484	79
GO:MF	Translation regulator activity	GO: 0045182	3.176178194325632e-8	46	19
GO:MF	Translation initiation factor activity	GO: 0003743	3.7891743887935486e-7	28	14
GO:MF	Coenzyme binding	GO: 0050662	0.000004504712288916028	186	37
GO:MF	Catalytic activity	GO: 0003824	0.000009193227505049144	3557	329
GO:MF	Electron transfer activity	GO: 0009055	0.00009413851727223974	40	14
GO:MF	NADH dehydrogenase activity	GO: 0003954	0.7672789560688666	10	4
GO:BP	Cellular amide metabolic process	GO: 0043603	2.1732985198865037e-25	327	90
GO:BP	Amide biosynthetic process	GO: 0043604	2.4948127365697987e-25	289	84
GO:BP	Organonitrogen compound biosynthetic process	GO: 1901566	3.602314663846417e-25	498	114
GO:BP	Peptide biosynthetic process	GO: 0043043	4.0045712627252803e-25	272	81
GO:BP	Peptide metabolic process	GO: 0006518	4.2857871078843195e-25	291	84
GO:BP	Translation	GO: 0006412	7.393454840335033e-25	268	80
GO:BP	Oxidation–reduction process	GO: 0055114	5.712176206243892e-12	534	94
GO:BP	Generation of precursor metabolites and energy	GO: 0006091	1.9288501323613227e-9	109	33
GO:BP	Small molecule metabolic process	GO: 0044281	5.014018036592118e-8	501	81
GO:BP	Organonitrogen compound metabolic process	GO: 1901564	4.0319711740266764e-7	1980	219
GO:CC	Intracellular	GO: 0005622	1.9616374270198e-54	1938	241
GO:CC	Cytoplasm	GO: 0005737	4.237751528306674e-42	700	133
GO:CC	Ribosome	GO: 0005840	6.638491755928224e-26	140	49
GO:CC	Intracellular nonmembrane-bounded organelle	GO: 0043232	1.887853402129643e-18	503	81
GO:CC	Nonmembrane-bounded organelle	GO: 0043228	1.887853402129643e-18	503	81
GO:CC	Intracellular organelle	GO: 0043229	3.1592018225889564e-18	1556	160
GO:CC	Organelle	GO: 0043226	2.813380227976983e-17	1603	161
GO:CC	Protein-containing complex	GO: 0032991	8.279406574286836e-12	922	102
GO:CC	Peptidase complex	GO: 1905368	5.728727766412518e-10	35	16
GO:CC	Endopeptidase complex	GO: 1905369	4.9033321759629505e-9	29	14

The adjusted enrichment *p* values with the number of proteins assigned to each subcategory are presented. Graphical representation is presented in Figure 2. Detailed representation and annotation of all proteins submitted to the analysis can be found in supplemental File S3.

Abbreviation: CC, celllular component.

### DRPs Establish a Complex Network of Interactions

**Fig. 5:**
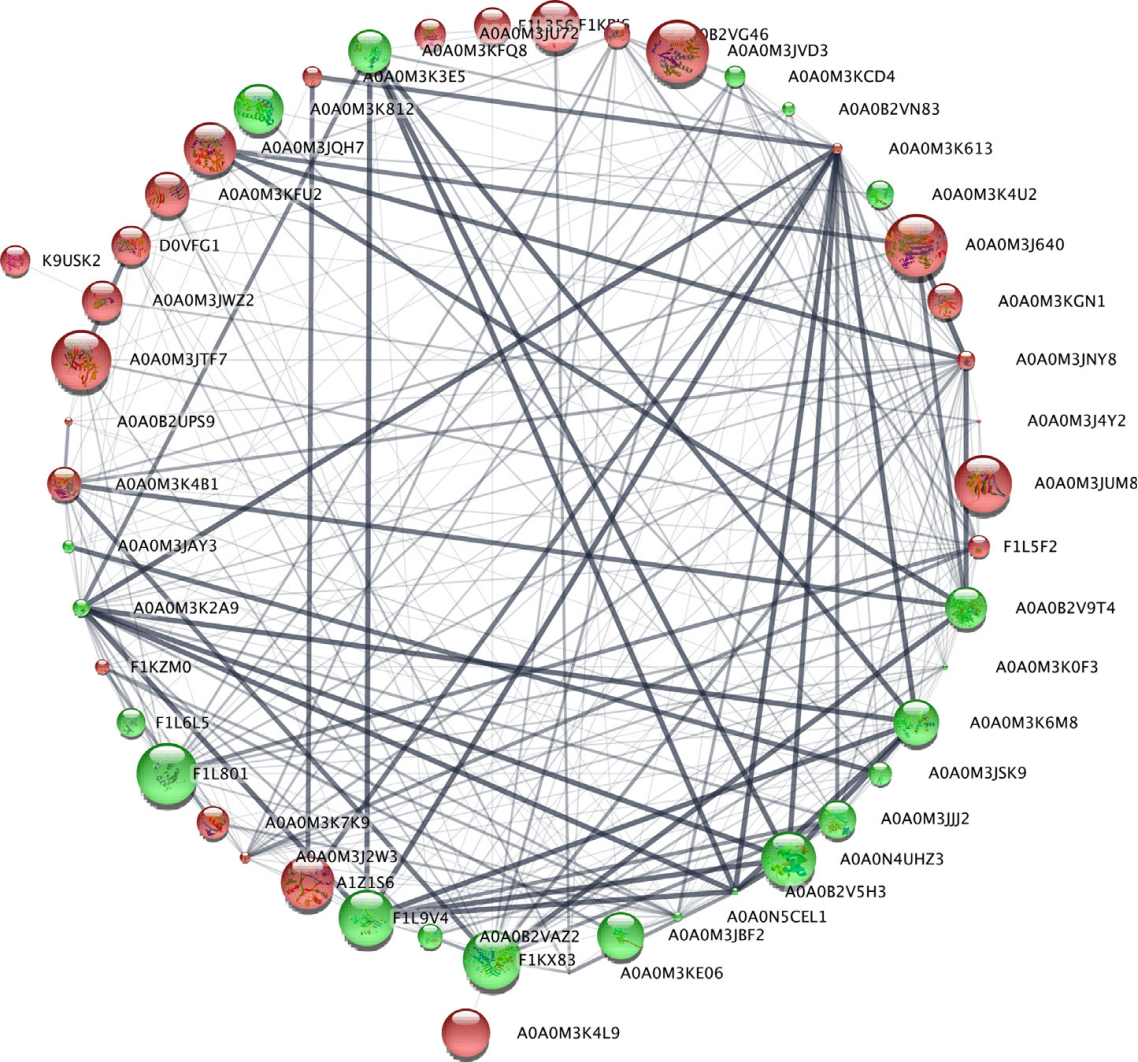
**Protein–protein interaction network analysis of differentially regulated proteins after treatment with LPS (0.2 μg/ml) in L3 larvae of *Anisakis simplex* s.s. performed in Cytoscape, version 3.8.0.** Downregulated proteins are marked in *red* and upregulated proteins in *green*. The lager the circle is, the smaller the *p* value was, which means that the protein modulation is more statistically significant. *Thick lines* indicate strong interactions. LPS, lipopolysaccharide.

**Fig. 6:**
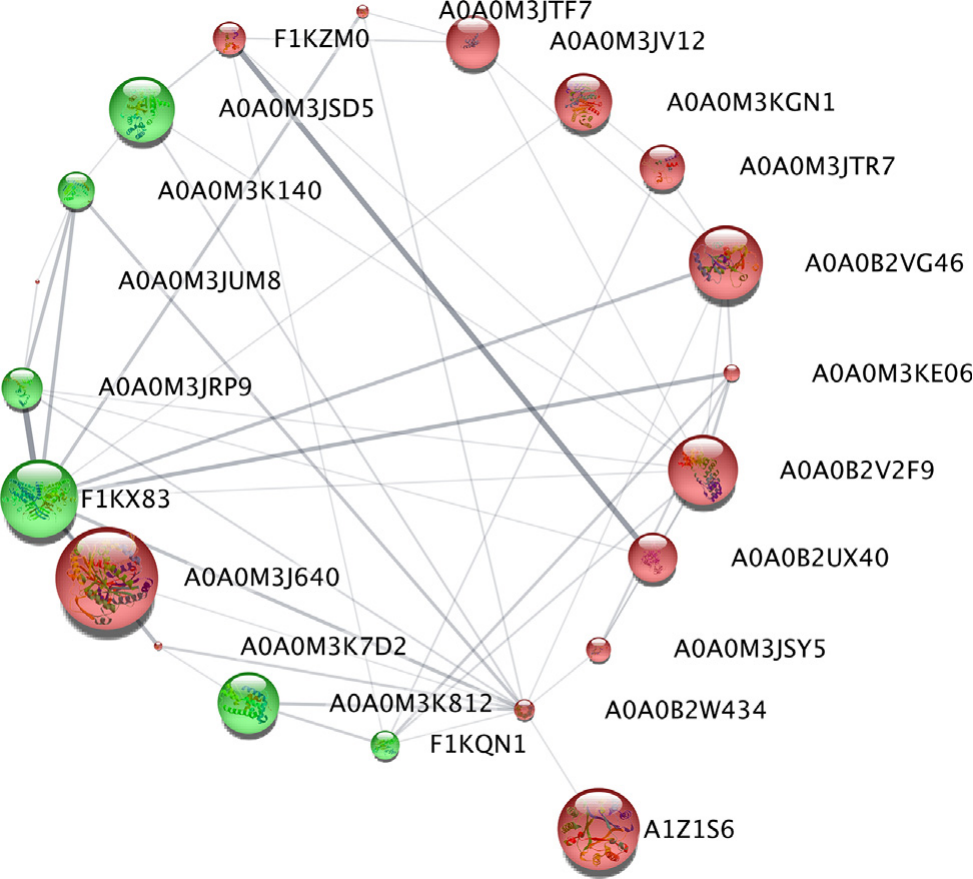
**Protein–protein interaction network analysis of differentially regulated proteins after treatment with LPS (1 μg/ml) in L3 larvae of *Anisakis simplex* s.s. performed in Cytoscape, versison 3.8.0.** Downregulated proteins are marked in *red* and upregulated in *green*. The larger the circle is, the smaller the *p* value was, which means that the protein modulation is more statistically significant. Thick lines indicate strong interactions. LPS, lipopolysaccharide.

The network of protein interactions was performed by submitting only DRPs (115 proteins) to Cytoscape (version 3.8.0.; NIGMS) and analyzed by stringApp (version 1.5.1.) (49). All interactions were shown in connection with coexpression, co-occurrence, and because of the appearance of any information on the interactions between those proteins in different databases (Figs. 5 and 6). The analysis demonstrated strong interaction networks (Figs. 5 and 6). According to the stringApp (version 1.5.1.), a total of 48 proteins constituted a very complex and strongly interactive network (275 interactions) at lower concentration of LPS (0.2 μg/ml [Fig. 5], whereas only 21 DRPs were metabolically related [50 interactions] at higher concentration of LPS [1 μg/ml]—[Fig. 6] (49)). The remaining input proteins not present in the database or not connected with any other protein were excluded from Figures 5 and 6. This mainly reflects the fact that those interactions have been identified on the background of *C. elegans*, a free-living species, not a parasitic one like *A. simplex* s.s, what was already discussed previously (36, 37).

Based on Reactome pathway analysis, the most complex nodes of the interactions were related to signal transduction (CEL-162582) and signaling by nuclear receptors (CEL-9006931) in a network constituted by DRPs after 1 μg/ml of LPS treatment (Fig. 6), whereas neutrophil degranulation (CEL-6798695), innate immune system (CEL-168249), immune system (CEL-168256), and translation (CEL-72766)—in a network constituted by DRPs after 0.2 μg/ml of LPS treatment (Fig. 5).

### Oxidative Stress Markers Confirm the Impact of LPS on *A. simplex* s.s.

**Fig. 7:**
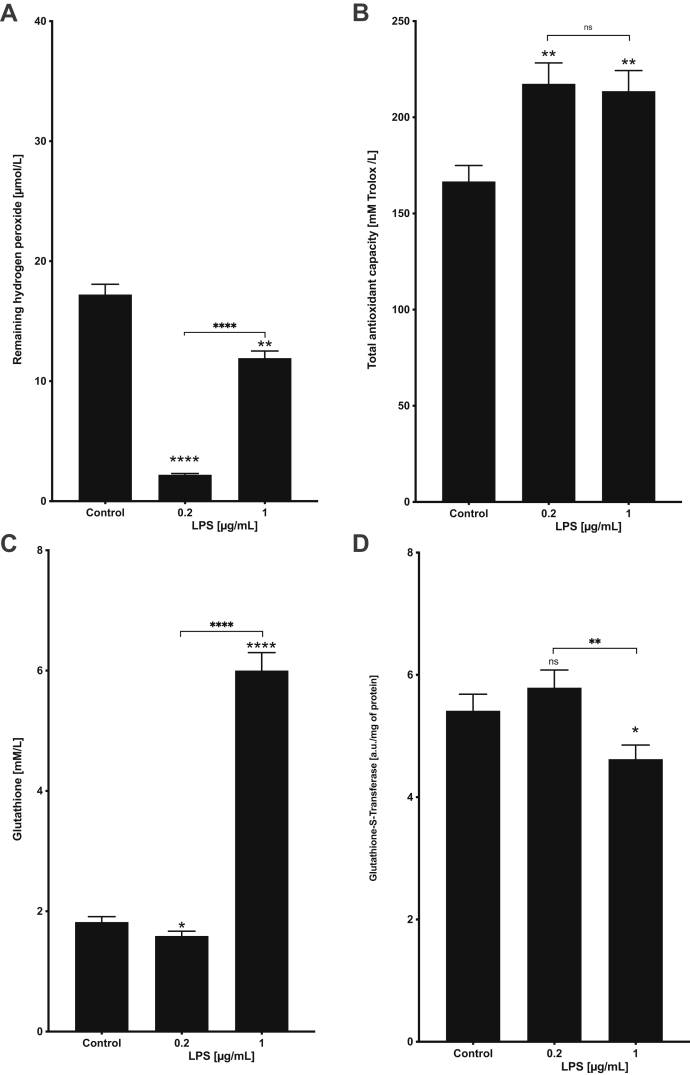
**Different biochemical indicators of oxidative stress in *Anisakis simplex* s.s. L3 larvae treated with different LPS concentrations.** Visualization of results of performed biochemical analyses: (*A*) peroxiredoxin activity, (*B*) total antioxidant capacity, (*C*) glutathione GSH content, and (*D*) glutathione S-transferase activity. All results were presented as mean ± SD (n = 3). Significant differences between LPS-treated samples and control samples are shown (*t* test), as well as between the treatments (*t* test). *p* Values were considered statistically significant, where 0.0332 (∗), 0.0021 (∗∗), 0.0002 (∗∗∗), and <0.0001 (∗∗∗∗). LPS, lipopolysaccharide.

The activity of PRDXs in the L3 larvae of *A. simplex* s.s. after stimulation by LPS was estimated by measuring the diminishment of H_2_O_2_ level by ferrous oxidation–xylenol orange assay compared with the control, where the lower quantity of remaining H_2_O_2_ indicates the higher activity of PRDXs (Fig. 7*A*). The activity of PRDXs in the larvae treated with 0.2 μg/ml of LPS was calculated as 2.2 μmol/l ± 0.11 remaining H_2_O_2_ (n = 3), whereas in the larvae treated with 1 μg/ml of LPS was calculated as 11.92 μmol/l ± 0.596 remaining H_2_O_2_ (n = 3). The activity of PRDXs in both groups of the LPS-treated larvae was significantly higher comparing to the control (17.22 μmol/l ± 0.86 remaining H_2_O_2_). Moreover, the activity of PRDXs was higher in the larvae treated with 0.2 μg/ml of LPS, than in those treated with 1 μg/ml of LPS.

The total antioxidant capacity was significantly higher in both LPS-treated groups of *A. simplex* s.s. larvae compared with the control (Fig. 7*B*). This also confirms the oxidative stress induction in the presented experimental setup.

The decreased content of the reduced form of GSH is an indicative of greater oxidative stress. In the larvae treated with 0.2 μg/ml of LPS, the GSH content was significantly lower compared with the control, which indicates GSH oxidation to protect cells by neutralizing ROS. In the larvae treated with 1 μg/ml of LPS, the GSH content was significantly higher compared with the control. This indicates its increased synthesis during the LPS treatment. GST activity was associated with the content of GSH, which is the substrate of this enzyme; the higher the activity of the enzyme, the lower the content of GSH. This was confirmed by a higher GST activity in the larvae treated with 0.2 μg/ml of LPS (Fig. 7, *C* and *D*).

### Gene Expression of Selected Markers of Oxidative Stress

**Fig. 8:**
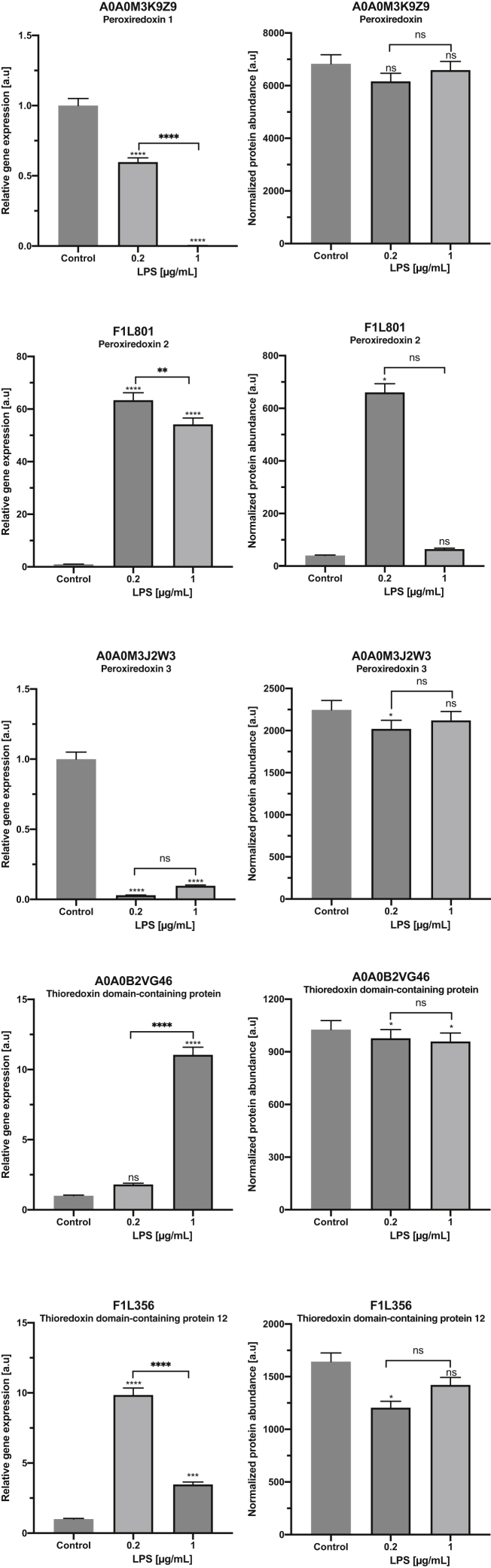
**The mRNA expression (*left*) and protein abundance (*right*).** The protein abundance was normalized between the samples and is originally from LC–MS/MS analysis. The transcription levels of selected genes encoding chosen DRPs were obtained from real-time PCR. The control is normalized to a value 1, and the graphs indicate the changes relative to the control. *p* Values were considered statistically significant, where 0.0332 (∗), 0.0021 (∗∗), 0.0002 (∗∗∗), and <0.0001 (∗∗∗∗). DRP, differentially regulated protein.

The results of real-time PCR showed that the mRNA levels of two genes (*prdx-2* and *prdx-3*) were approximately consistent with the protein levels in the larvae treated with LPS (LPS 0.2 or LPS 1). The *prdx-2* gene expression as well as its protein abundance were both upregulated in LPS 0.2 samples. Moreover, downregulation of *prdx-3* expression and protein abundance was noticed in both treatments compared with the control (Fig. 8). Nevertheless, the mRNA levels of *prdx-1* and both *thioredoxin domain–containing proteins* were inconsistent with the protein abundance levels.

## Discussion

The biome of the human body contains a diverse range of microorganisms named the microbiota, including bacteria, viruses, fungi, protozoa, and helminths (54). The prevalence of coinfection exceeds one-sixth of the global population (55). This concept is of considerable interest because microorganism interactions can affect host pathology and their own virulence evolution (56). Many factors can affect or change the relationship between commensal bacteria and intestinal parasites, such as competition for food resources (57), the secretion of bacterial growth inhibitors by certain species (58, 59), and host age and diet (15). The interaction between the host, intestinal parasites, and commensal bacteria should be studied, because of the fact that parasites can cause direct or indirect changes in the bacteria, and vice versa. At the same time, the latest hypothesis is that intestinal bacteria and intestinal parasites do not have to compete in the same environment. These interactions can be neutral, harmful, or have beneficial effects (15, 16). This crosstalk can be considered as a specific metabolic integration system (60). This hypothesis, however, has not been clearly proven and requires further research (28, 61).

In the present study, according to our knowledge, we explored for the first time the response of parasitic nematode, *A. simplex* s.s., to the treatment with LPS isolated from *E. coli*, a Gram-negative bacterium, which is frequently the first to colonize human infants and is a lifelong colonizer of adults (62). All Gram-negative bacteria are enclosed by an outer membrane, which acts as an additional protection barrier preventing the entry of toxic compounds including antibiotics and antimicrobial peptides (63). The outer membrane component, LPS plays a crucial role in the antimicrobial susceptibility of *E. coli* (64). LPS is one of the virulent determinants in *E. coli* that enhances its pathogenicity to provoke septicemia and resistance against antibiotics (64). Moreover, LPS is classified as thymus-independent type 1 antigen, which means that it can activate B cells without T-cell help (65). At the biochemical level, LPS is recognized by the Toll-like receptors preferentially expressed on phagocytes, dendritic cells, and epithelial cells at sites of bacterial entry to the host. However, it was confirmed that the existence of host responses to LPS does not require Toll-like receptor 4 (65, 66). Thus, it can be concluded that LPS is one of the first molecules responsible for the interaction with the host or with coexisting microorganism in the host's body, for example, helminth.

Therefore, to determine the response of *A. simplex* s.s. during interaction with LPS, the parasite proteome profile and activity of oxidative stress mediators in the larvae were analyzed.

According to literature data and our preliminary studies on the expression of *gdf 11* (homolog of human TGF-β), two doses of LPS have been selected for *in vitro* experiments. It is well known that helminths use the immunomodulatory ability of the TGF-β pathway to drive host cells to produce this cytokine and promote the long-term establishment of the parasite in mammalian tissues (40, 67). Literature data confirmed that helminths encode endogenous members of the TGF-β ligand and receptor family, which can interact with cognate partners of vertebrate origin (68, 69, 70, 71). Up to now, various zoonotic nematode species like *Ancylostoma caninum* and *Brugia malayi*, and trematodes such as *Fasciola hepatica*, are able to produce proteins (72), which mimic host's TGF-β by replicating its biological and functional properties. This in consequence abates inflammation *in vivo* (40). Therefore, in our *in vitro* model, we tested the response of *A. simplex* s.s. to LPS doses determined on the expression of the *A. simplex gdf 11* previously identified by Łopieńska-Biernat *et al.* (41) during genome-wide analysis of this species (Fig. 1*A*). The most significant changes in *gdf 11* expression after treatment with various doses of LPS prompted us to select two of them to design a proteomics experiment (0.2 and 1 μg/ml).

Proteomic analysis by LS–MS/MS revealed in a total 1148 proteins (supplemental File S2) in *A. simplex* s.s. L3 larvae treated with LPS. The results indicate that 115 proteins were differentially regulated in the LPS-treated larvae when compared with the controls (supplemental Tables S2 and S3). These proteins formed a very diverse group (supplemental Tables S2 and S3), including those with enzymatic, regulatory, and immunological properties, or those participating in oxidative stress response (Table 1).

The present results revealed that proteins regulating the immune metabolic pathways, described previously in *C. elegans*, have not been found in the larvae L3 of *A. simplex* s.s. However, we identified other proteins that can participate in the control of the immune response. For example, cathepsins act classically as lysosomal hydrolases that digest endogenous and exogenous endocytosed polypeptides (73), but in parasitic nematodes, they have primary roles in larval migration, molting, immune evasion, and in cleaving intact hemoglobin before it can be processed by other digestive peptidases. In our study, we identified cathepsin D (UniProt: D0VFG1) in *A. simplex* s.s. larvae after treatment with 0.2 μg/ml of LPS.

In addition, in the current study, we confirmed the presence of the macrophage migration inhibitory factor (MIF; UniProt: A1Z1S6) in the proteome of *A. simplex* s.s. after stimulation with LPS. MIF was one of the first cytokines to be discovered over 50 years ago (74). It was confirmed that Protozoa and parasitic nematodes secrete MIF that is structurally similar to human MIF (75, 76, 77). Parasitic MIF binds directly to the human MIF receptor CD74, activating the extracellular signal–regulated kinase pathway with immunomodulatory effects on variety of immune and epithelial cells. Recently, the studies on *A. ceylanicum* demonstrated that excess of parasite MIF partially displaces human MIF from its cognate receptor. It remains to be elucidated whether parasite MIF acts as an agonist, driving activation of downstream proinflammatory pathways, or as an antagonist, affecting CD74 in a nonproductive or an inhibitory fashion (78). Both scenarios could result in the modulation of the host immune response. It has been reported that MIF, isolated from *Haemonchus contortus*, decreased the production of tumor necrosis factor α, interleukin 1β (IL-1β) and IL-12p40, whereas it significantly increased the secretion of IL-10 and TGF-β in goat monocytes. In the same study, MIF-1 diminished the LPS-induced nitric oxide production *via* goat monocytes and the expression of major histocompatibility complex (MHC)-II on the monocytes (79). In our study, unexpectedly, the MIF protein expression was downregulated in the *A. simplex* larvae treated with both doses of LPS when compared with the control individuals (supplemental Tables S2 and S3). This could be caused by the presence/absence of some other signals from the host organism, which would trigger the parasite to modulate the host's immune system and evade immune defenses. Moreover, our experimental setup analyzed the proteomic response of the nematodes to the bacterial LPS. This also could be a reason for the downregulation of MIF expression protein in parasitic larvae. The simulation of *E. coli* coexistence with the parasitic larvae, within the host organism, and taking over by bacteria the modulation of host immunity, might cause the downregulation of the parasite mechanisms of host's immune system modulation. However, such conclusion should be validated in further experiments.

In presented cohort of the DRPs, we also found endoplasmic reticulum aminopeptidase 1 (ERAP1), which is primarily responsible for the generation of the antigenic repertoire presented by MHC-I molecules, in the control of susceptibility of different infectious diseases (80). ERAP1 is mainly responsible for the shaping of peptides through the antigen processing machinery origin in the cytoplasm by the proteasome that cuts intracellular proteins into heterogeneous fragments. Peptides are subsequently transported by the transporter associated with antigen processing within the endoplasmic reticulum, where they are trimmed at the N terminus, to generate suitable length peptides to be bound by MHC-I molecules and presented on cells' surface. In humans, ERAP1 and ERAP2 are the main aminopeptidases responsible for N-terminal peptide trimming (81). ERAP1, besides its significant function in acquired immunity, plays a key role in innate immune regulation. There is evidence that ERAP1 in the initial stages of pathogen recognition promotes shedding of cytokine receptors and nitric oxide formation, induces natural killer cells development and function, and triggers the phagocytic activity of macrophages (80). In our study, the ERAP1 expression was downregulated in the larvae treated with both doses of LPS compared with the controls (supplemental Tables S2 and S3), but the functions of this protein require more specific research like it was already explained in the previous example.

Nematodes, like other living organisms, have evolved physiological mechanisms to respond to different pathogens by activating specific gene expression and protein production (82). The current study revealed the presence of antioxidative proteins among various DRPs. There is evidence that parasite survival depends upon endogenous antioxidant defense system (26). To confirm the activity of this system in L3 larvae of *A. simplex* s.s. after LPS treatment, different biochemical analyses were carried out (Fig. 7). Herein, we described the activity of antioxidant proteins, PRDXs, in *A. simplex* s.s. The PRDXs, in prokaryotes as well as in eukaryotes, are considered to be the primary cellular guardians against oxidative stress by sensing and detoxifying H_2_O_2_. Although PRDXs have been initially thought to be much less efficient peroxide reductases than catalases and GSH peroxidases, it was proven that they reduce more than 90% of cellular H_2_O_2_ (83, 84). Nonetheless, their central role as peroxide scavenging enzymes among the cellular arsenal of antioxidant enzymes has been probably underestimated until recently (26, 85, 86).

In the group of upregulated proteins, obtained after LPS treatment, we identified PRDX-2 (F1L801), whereas PRDX-3 (A0A0M3JZW3) and two thioredoxin domain–containing proteins (A0A0B2VG46 and F1L356) were reported in the group of downregulated proteins (supplemental Tables S2 and S3). Interestingly, the PRDX-1 (A0A0M3K9Z9) was found in the group of not modulated proteins. Our gene expression data on PRDXs demonstrate similar pattern—downregulation of *prdx-1* and *prdx-3*, whereas upregulation of *prdx-2*, when compared with the control (Fig. 8). Xu *et al.* (87) reported a higher expression of *prdx-2* than *prdx-3* in developmental stages of *C. elegans*. It has been also reported that *prdx-2* from *C. elegans* is more important for protecting against H_2_O_2_ than *prdx-3* (88), what is consistent with our results (Fig. 8). Similarly, *prx-1* expression level in *Teladorsagia circumcincta* was decreased in L3 development stage (89). In addition, the presence of PRDX-1 in a group of not modulated proteins and its gene downregulation (Fig. 8) might directly indicate different PRDX isoform activities depending on the various stress conditions or the tissue type. For example, PRDX-1b in the adult *B. malayi* was localized in the hypodermis and lateral chord and was not secreted by/or at the surface of the larval or adult worms (90). In contrast, PRDX-1a in *Onchocerca volvulus* was found in the larval and adult hypodermis and cuticle and appears to be secreted by both (91, 92). The closely related homolog *Bm* PRDX-1a (90) could be likewise surface localized or secreted, as it is antigenic in mice (93) and humans (94, 95). The PRDX in pine wood nematode, *Bursaphelenchus xylophilus*, was broadly expressed across different tissues and could be secreted outside the nematode (96). In the present study, the gene expression of two *thioredoxin domain–containing proteins* (A0A0B2VG46 and F1L356) was upregulated compared with the control (Fig. 8). The expression of thioredoxins in *H. contortus* throughout the life cycle was also evaluated by quantitative real-time PCR. This study demonstrated that *trx-5* was expressed in third-stage larvae at levels like those of *trx-1* and *trx-3*, so in this case, the differentiation according to the isoform in a specific tissue has not taken place (97, 98). It should be highlighted that the mRNA levels of both *thioredoxin domain–containing proteins* in *A. simplex* s.s. were inconsistent with its protein's abundance level. In many studies, protein expression profile is inconsistent with gene expression level (99, 100). It should be emphasized that the regulatory processes occurring after mRNA synthesis can involve post-transcriptional and translational modifications. Moreover, the regulation of protein degradation is essential in controlling steady-state protein abundance; however, the translation efficacy is a single best predictor of protein levels (100). The global overview is that most mRNAs and proteins are stable unless genes need to respond quickly to the stimulus (99). All these aforementioned processes take place at a very fast pace, and thus, they may cause discrepancies at the level of gene and corresponding protein expression. However, in addition to proteomic methods, analysis of *A. simplex* larval mRNA expression can provide useful information on genes associated with parasite–human microbiome interactions. Gene expression profiles *versus* abundance of their proteins could provide clues to their direct role in facilitating parasite survival and their influence on parasite–human microbiome interactions, as well as parasite–host adaptations (41, 101, 102, 103, 104).

Summarizing, parasitic nematodes as anerobic organisms might use antioxidant homeostasis, and a fully functional antioxidant defense system is crucial for their survival situations. Therefore, it is highly possible that a knockout of any of these basic cell molecular components (*e.g.*, proteins regulating oxidative stress), medication, or immunization can lead to parasite death. However, those are significant cell safeguards in all organisms, and back-up mechanisms seem to exist for each kind of detoxification action. Because of this reality, focusing on one individual cell reinforcement compound may not be adequate to deliver the parasite defenseless.

It is also worth mentioning that most of the endoparasites have a much-reduced set of redox proteins than their free-living and plant-parasitic relatives (105). A different response to the bacterial LPS in *A. simplex* s.s. than in *C. elegans* may also indicate parasitic variability and the system of coexistence between parasite and gut bacteria.

## Conclusions

The successful development and survival of parasitic nematodes depends on their effective and flexible response to stress conditions, like harsh environment, inside their hosts. Since the life cycle of parasitic nematodes takes place entirely within host tissues, it is reasonable to assume that nematode exposure to microbiome is not uncommon, especially in the case of gastrointestinal nematodes.

Our findings indicate, for the first time, the complexity of the proteomic response of parasitic nematode, *A. simplex* s.s., to bacterial LPS. This experimental setup mimics the coexistence of helminth and gut bacteria in the host. The simulation of the crosstalk between parasitic nematode and bacteria showed us the complexity of the changes occurring in the parasite organism triggered by bacterial LPS *in vitro* and led us to conclude that the obtained results are hugely valuable in planning future strategies for studying helminths and can be a crucial step in the integrated systems biology approach to describe a relationship between parasite, host, and its commensal bacteria. In addition, understanding the interrelationships of microorganisms could reveal how such symbioses can shape a host organism's biology.

## Data Availability

Raw files (TMT-based proteomics of *A. simplex* s.s. in response to LPS; no. MSV000087010) are publicly and freely accessible from the MassIVE Repository (www.massive.ucsd.edu).

## Supplemental data

This article contains supplemental data.

## Conflict of interest

The authors declare no competing interests. The funders had no role in the design of the study; in the collection, analyses, or interpretation of data; in the writing of the article, or in the decision to publish the results.
