# Supplementary figures and images for: A Complex Proteomic Response of the Parasitic Nematode Anisakis simplex s.s. to Escherichia coliLipopolysaccharide
Source: Mol Cell Proteomics. 2021 Oct 19;20:100166. doi: 10.1016/j.mcpro.2021.100166 (PMC8605257; doi:10.1016/j.mcpro.2021.100166)

A

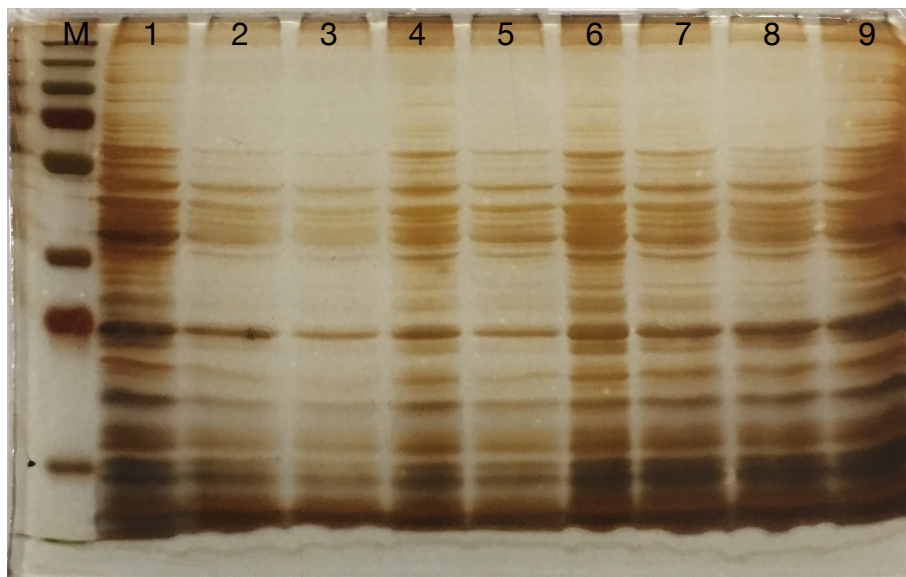

B

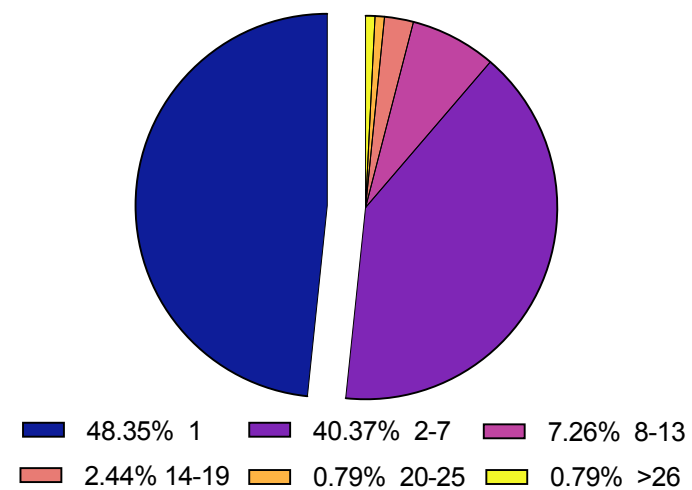

C

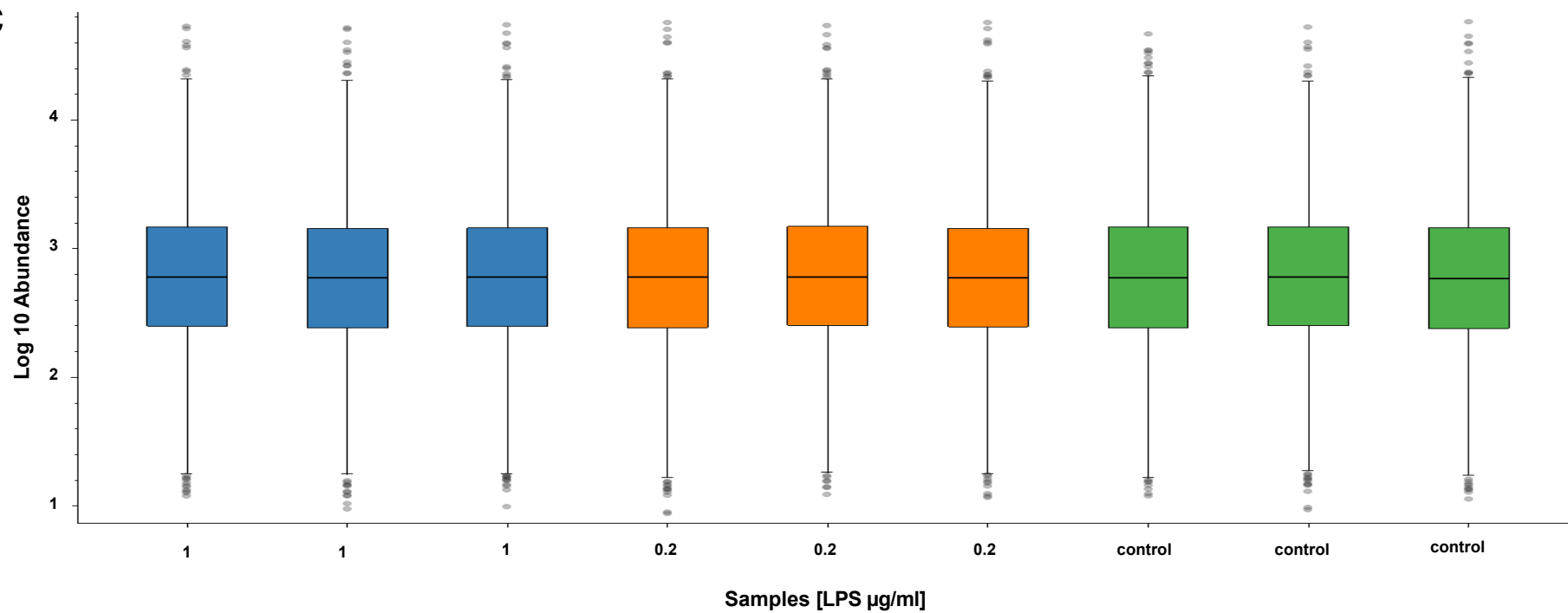

Supplement: Supplemental Figure S1 [file mmc1.pdf]

RT :0.00-252.00

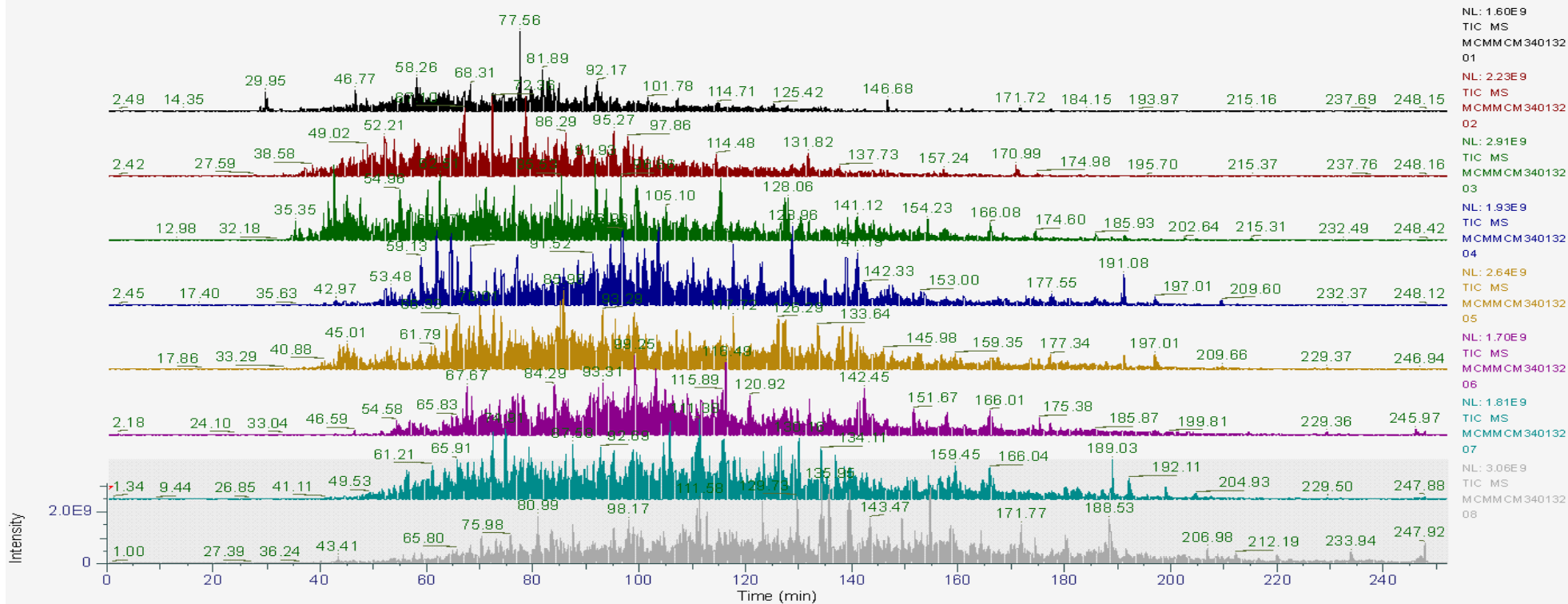

MCMCM34013204#19250 RT: 119.34 Av: 1 NL: 6.33E+004  
T: P TMS + p NSI d F u l m s 2 799.00 [3h04.00] [100.00-2000.00]

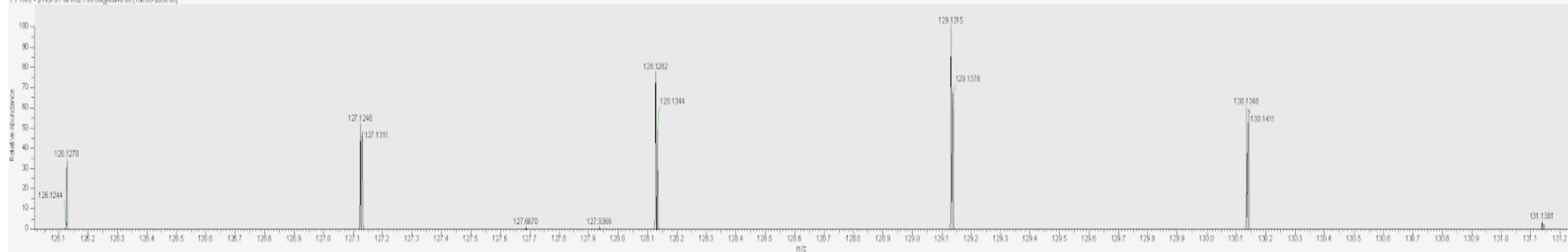

Supplement: Supplemental Figure S2 [file mmc2.pdf]
