# Supplementary material for: A Complex Proteomic Response of the Parasitic Nematode Anisakis simplex s.s. to Escherichia coliLipopolysaccharide
Source: Mol Cell Proteomics. 2021 Oct 19;20:100166. doi: 10.1016/j.mcpro.2021.100166 (PMC8605257; doi:10.1016/j.mcpro.2021.100166)
Supplement: Supplemental Figure S3 [file mmc3.pdf]

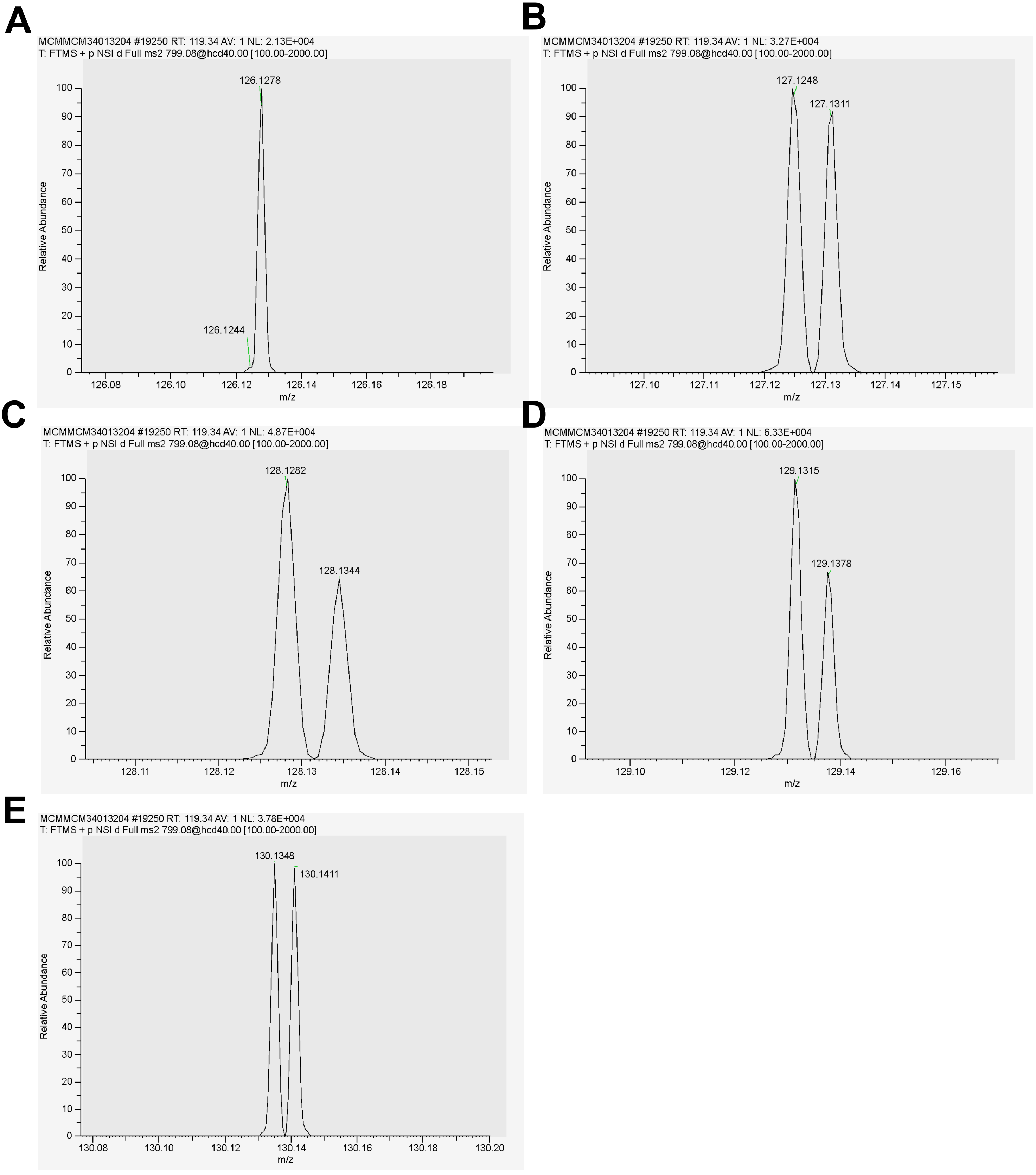
A
MCMMCM34013204 #19250 RT: 119.34 AV: 1 NL: 2.13E+004
T: FTMS + p NSI d Full ms2 799.08@hcd40.00 [100.00-2000.00]
126.1278
126.1244
Relative Abundance
m/z
B
MCMMCM34013204 #19250 RT: 119.34 AV: 1 NL: 3.27E+004
T: FTMS + p NSI d Full ms2 799.08@hcd40.00 [100.00-2000.00]
127.1248
127.1311
Relative Abundance
m/z
C
MCMMCM34013204 #19250 RT: 119.34 AV: 1 NL: 4.87E+004
T: FTMS + p NSI d Full ms2 799.08@hcd40.00 [100.00-2000.00]
128.1282
128.1344
Relative Abundance
m/z
D
MCMMCM34013204 #19250 RT: 119.34 AV: 1 NL: 6.33E+004
T: FTMS + p NSI d Full ms2 799.08@hcd40.00 [100.00-2000.00]
129.1315
129.1378
Relative Abundance
m/z
E
MCMMCM34013204 #19250 RT: 119.34 AV: 1 NL: 3.78E+004
T: FTMS + p NSI d Full ms2 799.08@hcd40.00 [100.00-2000.00]
130.1348
130.1411
Relative Abundance
m/z
