# Supplementary material for: A Complex Proteomic Response of the Parasitic Nematode Anisakis simplex s.s. to Escherichia coliLipopolysaccharide
Source: Mol Cell Proteomics. 2021 Oct 19;20:100166. doi: 10.1016/j.mcpro.2021.100166 (PMC8605257; doi:10.1016/j.mcpro.2021.100166)
Supplement: Supplemental Figure S4 [file mmc4.pdf]

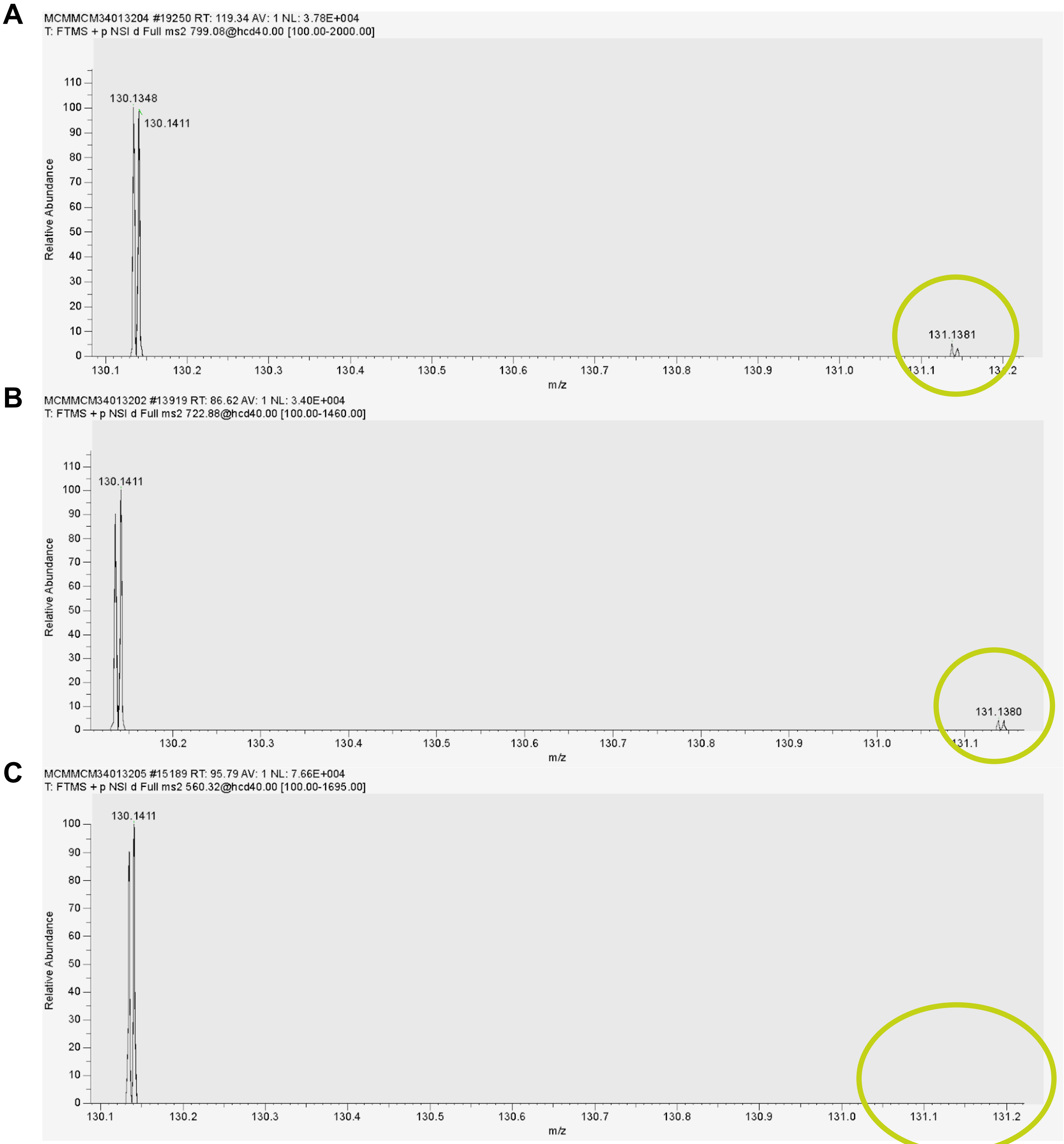

A
MCMMCM34013204 #19250 RT: 119.34 AV: 1 NL: 3.78E+004
T: FTMS + p NSI d Full ms2 799.08@hcd40.00 [100.00-2000.00]
130.1348
130.1411
131.1381
Relative Abundance
m/z
B
MCMMCM34013202 #13919 RT: 86.62 AV: 1 NL: 3.40E+004
T: FTMS + p NSI d Full ms2 722.88@hcd40.00 [100.00-1460.00]
130.1411
131.1380
Relative Abundance
m/z
C
MCMMCM34013205 #15189 RT: 95.79 AV: 1 NL: 7.66E+004
T: FTMS + p NSI d Full ms2 560.32@hcd40.00 [100.00-1695.00]
130.1411
Relative Abundance
m/z
