# Supplementary material for: A Complex Proteomic Response of the Parasitic Nematode Anisakis simplex s.s. to Escherichia coliLipopolysaccharide
Source: Mol Cell Proteomics. 2021 Oct 19;20:100166. doi: 10.1016/j.mcpro.2021.100166 (PMC8605257; doi:10.1016/j.mcpro.2021.100166)
Supplement: Supplemental Table S1 [file mmc8.docx]

Table S1. The list of primers used for Real-time PCR assay for determination of mRNA level of selected genes in *A. simplex* s.s. invasive larvae during LPS treatment.

| Gene name | Forward primer | Reverse primer |
| --- | --- | --- |
| Growth/differentiation factor 11  Actin  Peroxiredoxin 1  Peroxiredoxin 2  Peroxiredoxin 3  Thioredoxin domain-containing protein  Thioredoxin domain-containing protein 12 | ACGCAGTTCAACCGAGAAGT  TGGAGTGGTGCTTGACTCAG  AGGAGGACTTGGACCAATGC  TCTTGGCCCGATGCATATCC  TGGTGGCTTGTTCATGTGAT  GTCTGGTGGCTCGAAAAGAG  CGGCGATGACATCAATTGGG | TGCAATCGACATTCACCACT  TCACGAACAATCTCACGCTC  CAATTAGCTGGACAAACTTCTCC  CGGACCGATAAGCGATTCCA  GCATGGCGAATCATACCTTT  CGACCTCAGAAAGTGCATCA  GGTTCCTCCTTACCGACGAC |
