# Supplementary material for: A Complex Proteomic Response of the Parasitic Nematode Anisakis simplex s.s. to Escherichia coliLipopolysaccharide
Source: Mol Cell Proteomics. 2021 Oct 19;20:100166. doi: 10.1016/j.mcpro.2021.100166 (PMC8605257; doi:10.1016/j.mcpro.2021.100166)
Supplement: Supplemental Table S2 [file mmc9.docx]

Table S2. The list of differentially regulated proteins in *A. simplex* s.s. after treatment with 0.2 μg /mL of LPS (FC = 1.0; p-value ≤ 0.05).

| **Modulation** | **Accession No.** | **Protein names** | **Abundance Ratio:  (LPS 0.2) /(control)** | **Abundance Ratio  P-Value:  (LPS 0.2) / (control)** |
| --- | --- | --- | --- | --- |
| Up | A0A0M3J236 | 40S ribosomal protein S18 | 1.144 | 0.03468731 |
| Up | A0A0M3JD69 | 40S ribosomal protein S4 | 1.254 | 0.03917663 |
| Up | A0A0M3JRP9 | 60S acidic ribosomal protein P0 | 1.099 | 0.00090446 |
| Up | A0A0M3IZH8 | 60S ribosomal protein L13a | 1.259 | 0.03692347 |
| Up | A0A0M3KFV9 | 60S ribosomal protein L14 | 1.197 | 0.0223089 |
| Up | A0A0M3K1E5 | 60S ribosomal protein L27 | 1.15 | 0.02518416 |
| Up | A0A0M3K4R1 | 60S ribosomal protein L31 | 1.109 | 0.04927686 |
| Up | A0A0M3JBZ5 | 60S ribosomal protein L5 | 1.132 | 0.0173272 |
| Up | A0A0M3KAA6 | 60S ribosomal protein L9 | 1.198 | 0.04255133 |
| Up | A0A0M3J315 | ATP synthase subunit O. mitochondrial | 1.247 | 0.04829839 |
| Up | A0A0M3KAG0 | CCR4-NOT transcription complex subunit 7 | 1.065 | 0.03041969 |
| Up | A0A0M3JY95 | Dehydrogenase/reductase SDR family member 1 | 1.142 | 0.04084786 |
| Up | A0A0M3K8M6 | Dihydroorotate dehydrogenase (quinone) mitochondrial | 1.073 | 0.02994897 |
| Up | A0A0M3K0N6 | Dolichyl-diphosphooligosaccharide--protein glycosyltransferase subunit 1 | 1.078 | 0.02328687 |
| Up | A0A0M3JRE7 | Elongation factor Ts. mitochondrial (EF-Ts) (EF-TsMt) | 1.226 | 0.02359172 |
| Up | A0A0M3JVC5 | Elongation factor Tu homologue | 1.282 | 0.03139692 |
| Up | A0A0M3K507 | Gelsolin-like protein 1 | 1.146 | 0.01935123 |
| Up | A0A0M3JZV8 | Histone H2A | 1.137 | 0.03811038 |
| Up | A0A0M3K7C8 | Histone H2B | 1.205 | 0.00853395 |
| Up | A0A0M3J0S3 | Hydroxymethylglutaryl-CoA synthase | 1.201 | 0.0458994 |
| Up | A0A0M3JH96 | IF rod domain-containing protein | 1.816 | 0.00744473 |
| Up | A0A0M3IZR0 | L-2-hydroxyglutarate dehydrogenase. mitochondrial | 1.14 | 0.02606027 |
| Up | A0A0M3K812 | Letm1 RBD domain-containing protein | 1.123 | 0.01107992 |
| Up | A0A0M3KFQ8 | MPN domain-containing protein | 1.076 | 0.01674425 |
| Up | A0A0M3JBF2 | Myosin_tail_1 domain-containing protein | 1.051 | 0.01482464 |
| Up | A0A0M3JSH8 | Notchless protein homolog 1 | 1.146 | 0.03826574 |
| Up | F1L801 | Peroxiredoxin 2 (EC 1.11.1.15) | 16.35 | 0.00304389 |
| Up | A0A0M3J168 | PKS_AT domain-containing protein | 1.23 | 0.04881515 |
| Up | A0A0M3JSK9 | Prohibitin | 1.105 | 0.03352708 |
| Up | A0A0M3K8I9 | Properdin | 1.376 | 0.02195228 |
| Up | A0A0M3KBG0 | Protein mesh | 1.129 | 0.03834389 |
| Up | A0A0M3K0F3 | Protein-serine/threonine kinase (EC 2.7.11.-) | 1.36 | 0.04752897 |
| Up | A0A0M3JAY3 | Putative ATPase | 1.316 | 0.04178002 |
| Up | A0A0M3JQZ3 | S10_plectin domain-containing protein | 1.105 | 0.01256518 |
| Up | A0A0M3KCD4 | Thioredoxin glutathione reductase | 1.157 | 0.03424175 |
| Up | A0A0M3JP58 | Tr-type G domain-containing protein | 1.23 | 0.01972567 |
| Up | A0A0M3K4U2 | Tricarboxylate transport protein. mitochondrial | 1.301 | 0.0319652 |
| Up | A0A0M3JDV6 | Trifunctional enzyme subunit alpha. mitochondrial | 1.19 | 0.04766271 |
| Up | A9XBJ8 | UA3-recognized allergen (Fragment) | 1.944 | 0.00453553 |
| Up | A0A0M3K2A9 | Guanine nucleotide-binding protein subunit beta-2-like 1 | 1.042 | 0.03637042 |
| Up | F1L9V4 | 60S ribosomal protein L3 (Fragment) | 1.326 | 0.00777064 |
| Up | F1KX83 | Delta-1-pyrroline-5-carboxylate synthase | 1.248 | 0.00657396 |
| Up | F1L6L5 | Guanine nucleotide-binding protein G(Q) subunit alpha (Fragment) | 1.199 | 0.03033727 |
| Up | F1KPK4 | Protein sidekick | 1.672 | 0.0232423 |
| Up | A0A0N4UC60 | Inosine-5'-monophosphate dehydrogenase (IMP dehydrogenase) (IMPD) (IMPDH) (EC 1.1.1.205) | 1.149 | 0.03930249 |
| Up | A0A0N4UHZ3 | Ribosomal_L7Ae domain-containing protein | 1.099 | 0.00823558 |
| Up | A0A0N4VDQ9 | Aamy domain-containing protein | 1.158 | 0.02581775 |
| Up | A0A0N5CEL1 | J domain-containing protein | 1.426 | 0.04547162 |
| Up | A0A0B2V5H3 | 40S ribosomal protein S15 | 1.128 | 0.04735577 |
| Up | A0A0B2V538 | ATP-binding cassette sub-family B member 9 | 1.124 | 0.02820444 |
| Up | A0A0B2V9T4 | Glutamate dehydrogenase | 1.262 | 0.01755856 |
| Up | A0A0B2VAZ2 | Inosine-5'-monophosphate dehydrogenase | 1.124 | 0.03253546 |
| Up | A0A0B2VJK9 | Phosphatidylinositol phosphatase PTPRQ | 1.485 | 0.01540049 |
| Up | A0A0B2VN83 | Sodium/potassium-transporting ATPase subunit alpha | 1.089 | 0.04092174 |
| Down | K9USK2 | Hemoglobin (Fragment) | 0.774 | 0.02622044 |
| Down | A0A0M3JGR5 | ADF-H domain-containing protein | 0.929 | 0.00999385 |
| Down | A0A0M3K4B1 | Aldehyde dehydrogenase | 0.802 | 0.02565018 |
| Down | A0A0M3JQH7 | Aspartate aminotransferase | 0.809 | 0.01056981 |
| Down | A0A0M3K0V9 | BcDNA.GH10229 | 0.865 | 0.04375866 |
| Down | D0VFG1 | Cathepsin D-like aspartic protease | 0.834 | 0.02273606 |
| Down | A0A0M3IYB9 | Charged multivesicular body protein 1b | 0.854 | 0.01340724 |
| Down | A0A0M3JS17 | Clathrin interactor 1 | 0.874 | 0.00114316 |
| Down | A0A0M3K613 | Elongation factor 2 | 0.915 | 0.04210679 |
| Down | A0A0M3KGN1 | Epidermal retinol dehydrogenase 2 | 0.874 | 0.0255739 |
| Down | A0A0M3J726 | ERAP1_C domain-containing protein | 0.785 | 0.01367618 |
| Down | A0A0M3KFU2 | Galectin | 0.711 | 0.016592 |
| Down | A0A0M3JBP5 | GH13725p | 0.939 | 0.00224217 |
| Down | A0A0M3JVD3 | Glutathione S-transferase class-mu 28 kDa isozyme | 0.868 | 0.00113003 |
| Down | A0A0M3JWZ2 | LAMP family protein lmp-1 | 0.865 | 0.01952126 |
| Down | A0A0M3IYP6 | LD27216p | 0.786 | 0.01209795 |
| Down | A0A0M3J101 | Lipoyltransferase 1. mitochondrial | 0.827 | 0.02976099 |
| Down | A1Z1S6 | Macrophage migration inhibitory factor | 0.784 | 0.01055181 |
| Down | A0A0M3K031 | Mago-bind domain-containing protein | 0.809 | 0.03012333 |
| Down | A0A0M3JNY8 | Malate dehydrogenase | 0.898 | 0.0358389 |
| Down | A0A0M3IY35 | MICOS complex subunit MIC10 | 0.84 | 0.03895449 |
| Down | A0A0M3J770 | Nuclear anchorage protein 1 | 0.818 | 0.03755126 |
| Down | A0A0M3KE06 | Papilin | 0.924 | 0.04920334 |
| Down | A0A0M3JTF7 | Peptidase A1 domain-containing protein | 0.709 | 0.00536051 |
| Down | A0A0M3JEQ3 | Peptidase_M1 domain-containing protein | 0.788 | 0.04041926 |
| Down | A0A0M3J2W3 | Peroxiredoxin 3 (EC 1.11.1.15) | 0.9 | 0.04210341 |
| Down | A0A0M3J640 | Phosphopantothenate--cysteine ligase | 0.904 | 8.1704E-05 |
| Down | A0A0M3JL06 | Probable fumarate hydratase. mitochondrial | 0.872 | 0.04296358 |
| Down | A0A0M3K7K9 | Profilin | 0.941 | 0.025938 |
| Down | A0A0M3JU72 | Protein 4.1 homolog | 0.877 | 0.02611521 |
| Down | A0A0M3K3E5 | Protein Mo25 | 0.886 | 0.03470136 |
| Down | A0A0M3J4Y2 | Putative aspartyl aminopeptidase | 0.803 | 0.0487283 |
| Down | A0A0M3J8X8 | Pyr_redox_2 domain-containing protein | 0.847 | 0.0377259 |
| Down | A0A0M3K4L9 | Receptor expression-enhancing protein | 0.624 | 0.0147658 |
| Down | A0A0M3JUM8 | RNA-binding protein squid | 0.902 | 0.00672396 |
| Down | A0A0M3JRW6 | Saposin B-type domain-containing protein | 0.731 | 0.03027023 |
| Down | U1MLF6 | Autophagy-related protein 3 | 0.478 | 0.00717353 |
| Down | U1MRY1 | Hexokinase | 0.846 | 0.00569527 |
| Down | F1KZM0 | Myosin regulatory light chain | 0.846 | 0.04037311 |
| Down | F1L5F2 | Protein disulfide-isomerase 2 | 0.739 | 0.03398994 |
| Down | F1L356 | Thioredoxin domain-containing protein 12 | 0.734 | 0.02492296 |
| Down | F1KPJ6 | Transmembrane cell adhesion receptor mua-3 | 0.849 | 0.01083877 |
| Down | A0A0N5A872 | BAR domain-containing protein | 0.896 | 0.02880559 |
| Down | A0A0B2UPS9 | Putative medium-chain specific acyl-CoA dehydrogenase 1. mitochondrial | 0.883 | 0.04618951 |
| Down | A0A0B2VG46 | Thioredoxin domain-containing protein | 0.952 | 0.03222733 |
