# Supplementary material for: A Complex Proteomic Response of the Parasitic Nematode Anisakis simplex s.s. to Escherichia coliLipopolysaccharide
Source: Mol Cell Proteomics. 2021 Oct 19;20:100166. doi: 10.1016/j.mcpro.2021.100166 (PMC8605257; doi:10.1016/j.mcpro.2021.100166)
Supplement: Supplemental Table S3 [file mmc10.docx]

Table S3. The list of differentially regulated proteins in *A. simplex* s.s. after treatment with 1 μg/ mL LPS (FC = 1.0; p-value ≤ 0.05).

| **Modulation** | **Accession No.** | **Protein names** | **Abundance Ratio:  (LPS 1) / (control)** | **Abundance Ratio  P-Value:  (LPS 1) / (control)** |
| --- | --- | --- | --- | --- |
| Up | A0A0M3JRP9 | 60S acidic ribosomal protein P0 | 1,057 | 0,02362856 |
| Up | L7V3P8 | Ani s 12 allergen | 1,313 | 0,04433094 |
| Up | A0A0M3JSD5 | Arginine kinase | 1,097 | 0,01310005 |
| Up | A0A0M3K8S1 | B12-binding domain-containing protein | 1,112 | 0,0422318 |
| Up | F1KQN1 | Calcium-transporting ATPase (EC 7.2.2.10) | 1,135 | 0,03305228 |
| Up | F1KX83 | Delta-1-pyrroline-5-carboxylate synthase | 1,29 | 0,00794711 |
| Up | A0A0M3K812 | Letm1 RBD domain-containing protein | 1,096 | 0,01486373 |
| Up | A0A0M3K299 | Metalloendopeptidase (EC 3.4.24.-) | 1,441 | 0,02808151 |
| Up | A0A0M3K140 | Pyruvate dehydrogenase E1 component subunit alpha (EC 1.2.4.1) | 1,089 | 0,02456921 |
| Up | U1NBV5 | Succinate dehydrogenase [ubiquinone] flavoprotein subunit, mitochondrial | 1,108 | 0,01092923 |
| Up | A9XBJ8 | UA3-recognized allergen (Fragment) | 1,599 | 0,01770527 |
| Down | A0A0B2V2F9 | Ankyrin-2 | 0,916 | 0,01194945 |
| Down | A0A0B2W434 | Chaperonin-like protein Hsp-60, mitochondrial | 0,721 | 0,04610546 |
| Down | A0A0M3KGN1 | Epidermal retinol dehydrogenase 2 | 0,858 | 0,01757891 |
| Down | A0A0M3JSY5 | Laminin subunit beta-1 | 0,914 | 0,03528386 |
| Down | A1Z1S6 | Macrophage migration inhibitory factor | 0,747 | 0,00581425 |
| Down | A0A0M3K031 | Mago-bind domain-containing protein | 0,868 | 0,04563553 |
| Down | A0A0M3K7D2 | Mitochondrial-processing peptidase subunit alpha | 0,712 | 0,04955784 |
| Down | A0A0M3KA25 | MoCF_biosynth domain-containing protein | 0,877 | 0,0491517 |
| Down | F1KZM0 | Myosin regulatory light chain | 0,869 | 0,02736568 |
| Down | A0A0M3KE06 | Papilin | 0,928 | 0,04765809 |
| Down | A0A0M3J6S8 | Pepsin-I3 domain-containing protein | 0,861 | 0,03934832 |
| Down | A0A0M3JTF7 | Peptidase A1 domain-containing protein | 0,884 | 0,04850493 |
| Down | A0A0M3JEQ3 | Peptidase_M1 domain-containing protein | 0,823 | 0,02275075 |
| Down | A0A0M3J640 | Phosphopantothenate--cysteine ligase | 0,937 | 0,00058768 |
| Down | A0A0M3JUM8 | RNA-binding protein squid | 0,949 | 0,04968394 |
| Down | A0A0M3JTR7 | Tetraspanin | 0,892 | 0,02291488 |
| Down | A0A0B2VG46 | Thioredoxin domain-containing protein | 0,934 | 0,0096277 |
| Down | A0A0B2V4Y5 | Thyrotropin-releasing hormone-degrading ectoenzyme | 0,785 | 0,00843242 |
| Down | A0A0B2UX40 | Twitchin | 0,901 | 0,02136834 |
